# Nicotinamide mononucleotide promotes female germline stem cell proliferation by activating the H4K16ac-*Hmgb1*-*Fyn*-PLD signaling pathway through epigenetic remodeling

**DOI:** 10.1186/s13578-025-01387-w

**Published:** 2025-04-17

**Authors:** Hong Zhou, Yujie Liu, Geng G. Tian, Ji Wu

**Affiliations:** 1 Key Laboratory for the Genetics of Developmental and Neuropsychiatric Disorders (Ministry of Education), Bio-X Institutes, Shanghai Jiao Tong University, Shanghai, 200240 China; 2 School of Agriculture and Biology, Shanghai Jiao Tong University, Shanghai, 200240 China; 3 Key Laboratory of Fertility Preservation and Maintenance of Ministry of Education, School of Basic Medical Sciences, Ningxia Medical University, Yinchuan, 750004 China

**Keywords:** NMN, FGSCs, H4K16ac, *Hmgb1*, *Fyn*, PLD signaling pathway

## Abstract

**Background:**

Nicotinamide mononucleotide (NMN), an endogenous nucleotide essential for various physiological processes, has an unclear role and regulatory mechanisms in female germline stem cell (FGSC) development.

**Results:**

We demonstrate that NMN significantly enhances FGSC viability and proliferation. Quantitative acetylation proteomics revealed that NMN markedly increases the acetylation of histone H4 at lysine 16 (H4K16ac). Subsequent chromatin immunoprecipitation sequencing (ChIP-seq) and RNA sequencing (RNA-seq) identified high mobility group box 1 (*Hmgb1*) as a downstream target of H4K16ac, a finding further validated by ChIP-qPCR. Knockdown of *Hmgb1* reduced FGSC proliferation by disrupting cell cycle progression, inducing apoptosis, and decreasing chromatin accessibility. High-throughput chromosome conformation capture (Hi-C) analysis showed that *Hmgb1* knockdown induced A/B compartment switching, increased the number of topologically associating domains (TADs), and decreased chromatin loop formation in FGSCs. Notably, the chromatin loop at the promoter region of Fyn proto-oncogene (*Fyn*) disappeared following *Hmgb1* knockdown. ChIP-qPCR and dual-luciferase reporter assays further confirmed the interaction between *Hmgb1* and the *Fyn* promoter. Importantly, *Fyn* overexpression reversed the inhibitory effects of *Hmgb1* knockdown on FGSC proliferation. Proteomic analysis suggested this rescue was mediated through the phospholipase D (PLD) signaling pathway, as *Fyn* overexpression selectively enhanced the phosphorylation of PLD1 at threonine 147 without affecting serine 561. Furthermore, treatment with 5-fluoro-2-indolyldechlorohaloamide, a PLD inhibitor, nullified the pro-proliferative effects of *Fyn* overexpression.

**Conclusions:**

Our findings reveal that NMN promotes FGSC proliferation by activating the H4K16ac-*Hmgb1*-*Fyn*-PLD signaling pathway through epigenetic remodeling. These results deepen our understanding of FGSC proliferation and highlight potential therapeutic avenues for advancing FGSC applications in reproductive medicine.

**Supplementary Information:**

The online version contains supplementary material available at 10.1186/s13578-025-01387-w.

## Introduction

Nicotinamide mononucleotide (NMN) is a naturally occurring compound in the human body that plays a key role in the synthesis of intracellular nicotinamide adenine dinucleotide (NAD^+^) via various metabolic pathways [1]. In mammals, NMN is mainly produced from nicotinamide through the enzymatic action of nicotinamide phosphoribosyltransferase. Alternatively, NMN can be generated by the phosphorylation of nicotinamide riboside via nicotinamide riboside kinase, and subsequently converted into NAD^+^ by NMN adenylyltransferases [1, 2]. NMN has shown beneficial effects on conditions such as diet- and age-induced diabetes, recovery of mitochondrial function, heart and brain ischemia, vascular dysfunction, age-related physiological decline, obesity, DNA repair, colon degeneration, depression, and chronic hepatitis B [3–13]. Studies have demonstrated that NMN supplementation can enhance mitochondrial function, meiotic competency, fertilization rates, and embryonic development by restoring NAD^+^ levels, thus improving oocyte quality, especially in cases of maternal aging [14, 15]. Furthermore, Zhang et al. reported that NMN supplementation significantly increased sperm volume, density and motility, while reducing the proportion of abnormal sperm [16]. Additionally, research by Stein and Imai showed that NMN supports the maintenance of neural stem/progenitor cell populations during aging [17]. Moreover, Song et al. found that NMN promotes the self-renewal of mesenchymal stromal cells in aged mice by upregulating Sirtuin1 (SIRT1) [18]. Despite these findings, the role and regulatory mechanisms of NMN in germline stem cell development remain unclear.

In recent years, the rising incidence of infertility has increased the emphasis on protecting ovarian function and preserving female fertility. The discovery and successful establishment of female germline stem cell (FGSC) lines have opened up promising new approaches to treating infertility, understanding human oogenesis, and preserving fertility [19, 20]. A growing body of research has confirmed the existence of FGSCs in the ovaries of various species, including mice, rats, pigs, cattle, sheep, monkeys, and humans, and demonstrated their isolation and long-term culture in vitro [19–26]. FGSCs possess the ability to self-renew and differentiate into oocytes in vitro [19–23]. Moreover, when combined with gonadal somatic cells, FGSCs can form ovarian organoids [27, 28]. Given NMN’s established importance in a variety of physiological processes and its role in other stem cell populations, investigating its potential regulatory mechanisms in FGSC development is warranted.

Currently, a key research focus is on enhancing the proliferation efficiency of FGSCs to better understand their role in oogenesis, the restoration of ovarian function, and the maintenance of female fertility. Cell proliferation is a complex process requiring precise regulation of gene expression and signaling pathways. Epigenetic regulation plays a critical role in cell proliferation through mechanisms such as histone modifications, non-coding RNAs, DNA methylation, and chromatin remodeling, which all influence gene expression [29–32]. Additionally, key transcription factors regulate genes associated with the cell cycle, thereby controlling cell proliferation [33]. Cell signaling pathways, including the mitogen-activated protein kinase (MAPK), phosphatidylinositol-3-kinase (PI3K)/Akt, and Wingless/Integrated (Wnt)/β-catenin pathways, are also essential for cell proliferation and survival [29, 34–36]. Given the potential of NMN to enhance oocyte quality and support fertility preservation, it is crucial to understand how NMN influences FGSC proliferation.

In this study, we found that NMN significantly enhanced FGSC viability and proliferation, accompanied by increased acetylation of histone H4 at lysine 16 (H4K16ac). Chromatin immunoprecipitation sequencing (ChIP-seq) and RNA sequencing (RNA-seq) identified high mobility group box 1 (*Hmgb1*) as a downstream target of H4K16ac. Knockdown of *Hmgb1* inhibited FGSC proliferation by disrupting cell cycle progression and inducing apoptosis, as well as reducing chromatin accessibility and altering 3D chromatin structure. Further analyses, including assay for transposase-accessible chromatin sequencing (ATAC-seq), high-throughput chromosome conformation capture (Hi-C), and RNA-seq, revealed that Fyn proto-oncogene (*Fyn*) is a downstream target of *Hmgb1*. Overexpression of *Fyn* restored the FGSC proliferation that had been suppressed by *Hmgb1* knockdown. This rescue was mediated through the phospholipase D (PLD) signaling pathway, specifically by enhancing the phosphorylation of PLD1 at threonine 147 (Thr147). The pro-proliferative effect of *Fyn* overexpression was nullified by 5-fluoro-2-indolyldechlorohaloamide (FIPI), a PLD inhibitor. These findings provide valuable insights into the regulatory mechanisms underlying FGSC proliferation and suggest a new therapeutic target for female infertility.

## Materials and methods

### Chemical compound

β-NMN (N3501, Sigma-Aldrich) was dissolved in phosphate-buffered saline (PBS) at 100 mM and then diluted with medium to the desired concentrations. MC4033 (HY-149302, MCE) was prepared in dimethyl sulfoxide (DMSO) at 50 mM and diluted with medium as needed. FIPI (HY-12807, MCE) was dissolved in DMSO at 10 mM and similarly diluted before use.

### Animals

Female C57BL/6 mice were purchased from SLAC Laboratory Animal Co., Ltd (Shanghai, China). All animal experiments were approved by the Institutional Animal Care and Use Committee of Shanghai and conducted in strict compliance with the National Research Council Guide for the Care and Use of Laboratory Animals (Mechanisms of female germline stem cell development and ovarian function remodeling, 201703004).

### Isolation and culture of FGSCs

Mouse FGSC lines were isolated and cultured following established protocols [19, 27, 37]. Briefly, 6-day-old female C57BL/6 mice were euthanized using carbon dioxide. Ovaries were collected in ice-cold Hank’s balanced salt solution (HBSS) without calcium or magnesium, and cells were isolated through a two-step enzymatic digestion method. After initial culture, FGSCs were purified using DDX4-based immunomagnetic beads. The purified FGSCs were then maintained on mitomycin-treated STO (SIM mouse embryo-derived thioguanine- and ouabain-resistant) feeder cells at 37 °C with 5% CO_2_. The culture medium consisted of Minimum Essential Medium Alpha (12000022, Gibco), supplemented with 10% fetal bovine serum (FBS03ES-5001, Front), 10 ng/mL human basic fibroblast growth factor (10018b, PeproTech), 10 ng/mL murine epidermal growth factor (31509, PeproTech), 10 ng/mL murine glial-derived neurotrophic factor (45044, PeproTech), 10 ng/mL murine leukemia inhibitory factor (sc-4378, Santa Cruz Biotechnology), 1 mM MEM non-essential amino acids (11140050, Gibco), 1 mM sodium pyruvate (11360070, Gibco), 2 mM l-glutamine (A2916801, Gibco), 0.1 mM β-mercaptoethanol (M3148, Sigma-Aldrich), 100 IU/mL penicillin (15140163, Gibco), and 100 μg/mL streptomycin (15140163, Gibco). The medium was replaced every 48 h, and cells were passaged every 3–4 days at 1:3 or 1:5 ratio.

### Cell viability assay

Cell viability was measured using the Cell Counting Kit-8 (CCK-8, C0038, Beyotime) following the manufacturer’s instructions. Briefly, FGSCs were seeded at 5000 cells per well in a 96-well plate and cultured overnight. After treatment with different NMN concentrations for 24 or 48 h, 10 μL of CCK-8 reagent was added to each well for a 2-h incubation. The optical density at 450 nm was then measured with a multimode microplate reader (Spark, Tecan, Switzerland).

### Cell proliferation assay

Cell proliferation was measured using the Cell-Light EdU Apollo567 in vitro kit (C10310-1, RiboBio) following the manufacturer’s protocol. Briefly, FGSCs were seeded at 10,000 cells per well in a 48-well plate and cultured overnight. After treatment with different NMN concentrations for 24 or 48 h, 50 μM 5-ethynyl-2′-deoxyuridine (EdU) solution was added to each well for a 2-h incubation. Cells were then fixed with 4% paraformaldehyde for 30 min, neutralized with 2 mg/mL glycine for 5 min, and permeabilized with 0.5% Triton X-100 for 10 min. The cells were stained with Apollo for 30 min, counterstained with Hoechst 33342 for 10 min, and visualized under a fluorescent microscope (DMI3000B, Leica, Germany).

### Western blotting

Cells were lysed in RIPA buffer (P0013B, Beyotime) with a protease inhibitor cocktail (P1010, Beyotime). 10 µg of protein was separated by SDS-PAGE and transferred onto a PVDF membrane. After blocking with 5% skim milk for 1 h at room temperature, the membrane was incubated overnight at 4 °C with primary antibodies, followed by a 1-h incubation with HRP-conjugated secondary antibodies at room temperature. Protein bands were detected using ECL reagents and visualized with a chemical imaging system (Fusion FX, VILBER, France). The antibodies and their dilution factors were as follows: GAPDH (1:50,000, 60004–1-Ig, Proteintech), SIRT1 (1:2000, 13161-1-AP, Proteintech), H3 (1:2000, 17168-1-AP, Proteintech), anti-acetyllysine (1:2000, PTM-101, PTM Biotech), H4 (1:4000, 16047-1-AP, Proteintech), H4K16ac (1:1000, 13534S, CST), HMGB1 (1:1000, PA1-16926, Invitrogen), Cyclin D1 (1:4000, ab134175, Abcam), Cyclin E1 (1:1000, 11554-1-AP, Proteintech), Cyclin A2 (1:2000, ab181591, Abcam), Cyclin-dependent kinase 4 (CDK4; 1:2000, 11026-1-AP, Proteintech), Cleaved Caspase-3 (1:1000, 9664S, CST), Bcl-2-associated X protein (BAX; 1:1000, 2772T, CST), B-cell lymphoma 2 (BCL2; 1:1000, 3498T, CST), poly(ADP-ribose)polymerase (PARP; 1:1000, 9542T, CST), FYN (1:1000, ab125016, Abcam), FLAG (1:1000, F1804, Sigma-Aldrich), PLD1 (1:1000, 3832S, CST), p-PLD1 (Thr147) (1:1000, 3831S, CST), p-PLD1 (Ser561) (1:1000, 3834S, CST).

### Quantitative acetylation-modified proteome sequencing

Proteomics analysis was conducted by PTM Biotech Co., Ltd (Hangzhou, China). Briefly, proteins were extracted from cells using a lysis buffer, digested with trypsin overnight, and dissolved in an immunoprecipitation buffer. The supernatant was incubated with pre-washed acetylation resin at 4 °C overnight. Resin-bound peptides were washed three times, eluted, and then vacuum freeze-dried. After drying, salts were removed using C18 Zip Tips, and the samples were vacuum freeze-dried again for liquid mass spectrometry analysis.

### Chromatin immunoprecipitation followed by high-throughput sequencing (ChIP-seq) and ChIP-qPCR

Chromatin immunoprecipitation (ChIP) was performed using the Pierce Magnetic ChIP Kit (26157, Thermo Scientific) following the manufacturer’s instructions. Briefly, cells were cross-linked with 1% paraformaldehyde for 10 min at room temperature, then quenched with glycine solution for 5 min. The cells were resuspended in lysis buffer and sonicated to shear the chromatin. The lysate was incubated overnight at 4 °C with 5 μg of antibody (HMGB1, PA1-16926, Invitrogen; H4K16ac, 07-329, Sigma), followed by the addition of 20 μL of protein A/G magnetic beads. After washing and elution, the purified DNA was used for both ChIP-seq and ChIP-qPCR. ChIP-seq and data analysis were conducted by GENEFUND Biotech Co., Ltd (Shanghai, China). Primers sequences for ChIP-qPCR are listed in Table S1.

### RNA sequencing (RNA-seq)

Total RNA was extracted using TRIzol reagent (15596026, Invitrogen) following the manufacturer’s protocol. RNA quality and quantity were assessed with a NanoDrop 2000 spectrophotometer (Thermo Scientific, USA), and integrity was evaluated using an Agilent 2100 Bioanalyzer (Agilent Technologies, USA). Library preparation was performed with the TruSeq Stranded mRNA LT Sample Prep Kit (Illumina, USA). Transcriptome sequencing and analysis were conducted by OE Biotech Co., Ltd (Shanghai, China).

### Quantitative real-time PCR (qRT-PCR)

Total RNA was extracted with TRIzol reagent, and cDNA was synthesized using a reverse transcription kit (11141ES60, Yeasen). qRT-PCR was performed on an Applied Biosystems 7500 Real-Time PCR System (7500, Applied Biosystems, USA) using Hieff UNICON^®^ Universal Blue qPCR Master Mix (11184ES08, Yeasen). Cycling conditions were: 50 °C for 2 min, 95 °C for 2 min, followed by 40 cycles of 95 °C for 10 s and 60 °C for 30 s. Data were analyzed using the 2^−ΔΔCt^ method. Primers sequences for qRT-PCR are listed in Table S2.

### Lentiviral infection

Lentiviral vectors for *Hmgb1* knockdown or *Fyn* overexpression were prepared by OBiO Biotech Co., Ltd (Shanghai, China). FGSCs at 50% confluency were infected at a multiplicity of infection (MOI) of 50. After 24 h, the medium was replaced with fresh medium, and stable lines were selected using 2 μg/mL puromycin or 4 μg/mL blasticidin. *Hmgb1* shRNA sequences are listed in Table S3.

### Cell cycle analysis

Cell cycle analysis was performed with the Cell Cycle and Apoptosis Detection Kit (C1052, Beyotime) following the manufacturer’s instructions. Briefly, cells were trypsinized, washed with cold PBS, and fixed in cold 70% ethanol at 4 °C overnight. The next day, cells were washed again with cold PBS and incubated in propidium iodide staining solution at 37 °C for 30 min. Cell cycle distribution was analyzed using flow cytometry (CytoFLEX S, Beckman Coulter, USA) and FlowJo software.

### Apoptosis analysis

Apoptosis analysis was assessed using the eBioscience™ Annexin V Apoptosis Detection Kit (88-8007-72, Invitrogen) following the manufacturer’s instructions. Briefly, cells were trypsinized, washed with PBS, and then resuspended with binding buffer. Cells were incubated in binding buffer with 5 μL of fluorochrome-conjugated annexin V for 15 min at room temperature, followed by an additional wash in binding buffer and incubation with propidium iodide staining solution. Apoptosis was assessed using flow cytometry and analyzed with FlowJo software.

### Assay for transposase-accessible chromatin with high-throughput sequencing (ATAC-seq)

Cells were lysed using cold lysis buffer (10 mM Tris–HCl, pH 7.4, 10 mM NaCl, 3 mM MgCl₂, and 0.1% IGEPAL CA-630). Nuclei were isolated by centrifugation at 500×*g* for 10 min at 4 °C, and the supernatant was discarded. The nuclei pellet was resuspended in a transposition mix containing 25 μL of 2× TD buffer, 2.5 μL of transposase (S604-02, Vazyme), and 22.5 μL of nuclease-free water, followed by a transposition reaction at 37 °C for 30 min. After transposition, the sample was purified using the MinElute PCR Purification Kit (28006, Qiagen). The purified DNA was then used for ATAC-seq, which was conducted by GENEFUND Biotech Co., Ltd (Shanghai, China).

### High-throughput chromosome conformation capture (Hi‑C)

In situ Hi-C was conducted following established protocols [38]. Briefly, cells were cross-linked, lysed, and digested with the restriction enzyme MboI (R0147, NEB). Biotin was incorporated into the cohesive ends of DNA fragments prior to proximity ligation using T4 DNA ligase. After ligation, the DNA was purified and sheared into smaller fragments. Subsequent steps, including end repair, adenylation, and adapter ligation, were conducted using the NEBNext End Repair Kit (E6050, NEB). Biotin-labeled DNA fragments were captured using MyOne Streptavidin T1 beads (65602, Invitrogen). The Hi-C libraries were amplified using the KAPA HiFi Library Amplification Kit (KK2620, KAPA Biosystems), and size selection of the DNA fragments was performed using AMPure XP beads (A63880, Beckman Coulter).

### Dual-luciferase reporter assay

The targeted interaction between *Hmgb1* and the *Fyn* promoter region was assessed using the Dual Luciferase Reporter Gene Assay Kit (11402ES60, Yeasen). Briefly, 3T3 cells were cultured in 24-well plates for 24 h, followed by plasmid transfection using Lipo8000 (C0533, Beyotime). For the control group, pcDNA3.1, *Fyn*, and pRL-TK plasmid were transfected, while the *Hmgb1* overexpression group received pcDNA3.1-*Hmgb1*, *Fyn*, and pRL-TK plasmid. After 24 h, cell lysates were collected, and luciferase activities of Firefly and Renilla were measured using a multimode microplate reader (Spark, Tecan, Switzerland). Renilla luciferase activity was used as an internal control for data analysis. The primer sequences for plasmid construction are listed in Tables S4 and S5.

### Proteome sequencing

Proteomic analysis was conducted by PTM Biotech Co., Ltd (Hangzhou, China). Briefly, proteins were extracted from cells using a lysis buffer and digested with trypsin overnight. The resulting peptides were then freeze-dried under vacuum for liquid mass spectrometry analysis.

### Statistical analysis

Data are presented as the mean ± SEM. Statistical significance was determined using an unpaired two-tailed Student’s *t* test in GraphPad Prism 10 software (GraphPad Software, USA), with a *p* value <0.05 considered statistically significant.

## Results

### NMN enhances cell viability and proliferation of FGSCs

**Fig. 1:**
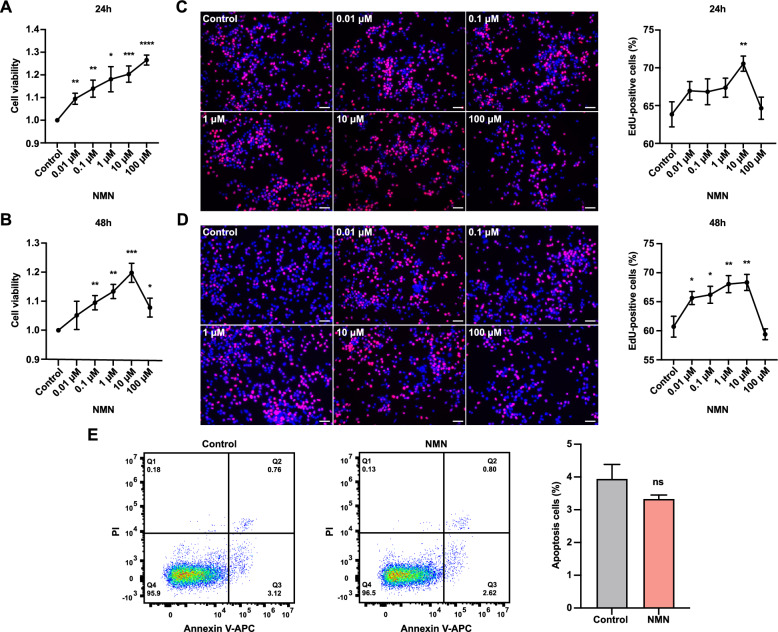
NMN enhances cell viability and proliferation of FGSCs. **A** Cell viability after 24-h treatment with different NMN concentrations. **B** Cell viability after 48-h treatment with varying NMN concentrations. **C** Representative EdU staining images and the percentage of EdU-positive cells in FGSCs treated with different NMN concentrations for 24 h. **D** Representative EdU staining images and the percentage of EdU-positive cells in FGSCs treated with varying NMN concentrations for 48 h. **E** Flow cytometry analysis showing apoptosis rates in control and NMN-treated groups. * *p* < 0.05, ** *p* < 0.01, *** *p* < 0.001, **** *p* < 0.0001, ^ns^ *p* > 0.05. Scale bars: 50 μm

To explore the role of NMN in regulating FGSC development, we treated FGSCs in vitro with different NMN concentrations for 24 and 48 h. CCK-8 assays showed that after 24 h of treatment, NMN significantly enhanced FGSC viability in a dose-dependent manner, with the most pronounced effect at 100 μM (Fig. 1A). After 48 h of treatment, NMN also promoted FGSC viability, but the effect varied; viability initially increased before decreasing at higher concentrations, peaking at 10 μM (Fig. 1B).

We further investigated the effect of NMN on FGSC proliferation. EdU assays revealed that after 24 h of treatment, NMN enhanced FGSC proliferation, with the strongest effect at 10 μM (Fig. 1C). After 48 h of treatment, NMN similarly promoted FGSC proliferation, with a rise followed by a decline as concentration increased, again peaking at 10 μM (Fig. 1D).

Next, we examined the impact of NMN on apoptosis in FGSCs. Flow cytometric analysis indicated that NMN treatment inhibited FGSC apoptosis, primarily affecting the early stages (Fig. 1E). These findings indicate that NMN treatment enhances cell viability and proliferation of FGSCs.

### NMN promotes H4K16 acetylation in FGSCs

**Fig. 2:**
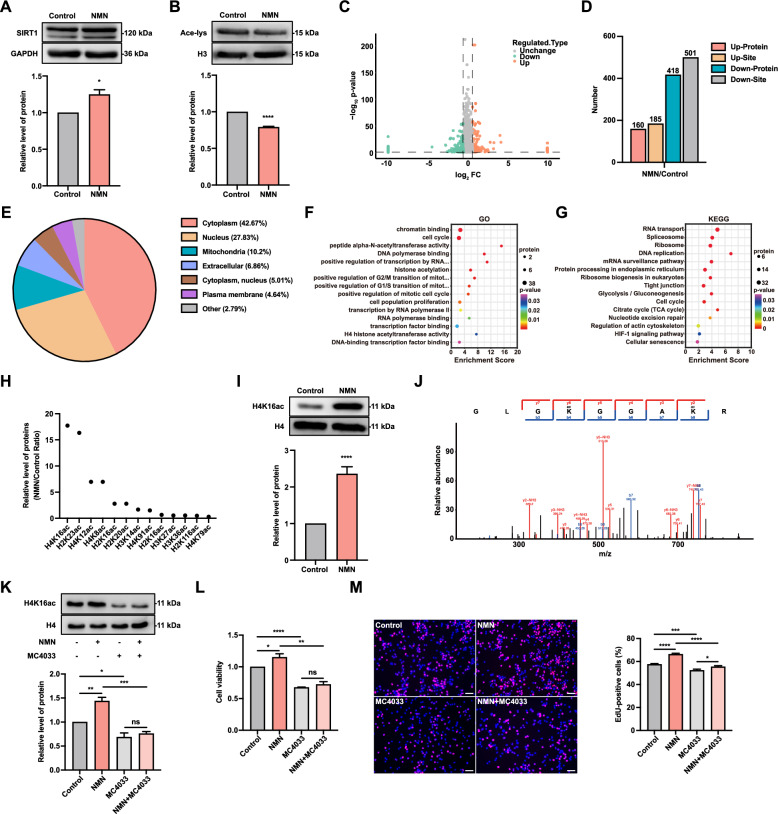
NMN promotes H4K16 acetylation in FGSCs. **A** Western blot analysis of SIRT1 expression in control and NMN-treated groups. **B** Western blot showing histone acetylation levels in control and NMN-treated groups. **C** Volcano plot of DEAPs between control and NMN groups. **D** Counts of DEAPs and DEASs between control and NMN groups. **E** Subcellular localization of DEAPs in control and NMN-treated groups. GO (**F**) and KEGG pathway (**G**) enrichment analyses of DEAPs. **H** Differentially acetylated histone modification sites in core histones. **I** Western blot analysis of H4K16ac levels. **J** Secondary mass spectrometry analysis of H4K16ac following NMN treatment. **K** Co-treatment with MC4033 reduces NMN-induced H4K16ac protein expression. **L** Cell viability following co-treatment with MC4033. **M** Representative EdU staining images and the proportion of EdU-positive cells after combined treatment with MC4033. * *p* < 0.05, ** *p* < 0.01, *** *p* < 0.001, **** *p* < 0.0001, ^ns^ *p* > 0.05. Scale bars: 50 μm

To explore the regulatory mechanisms by which NMN affects FGSC development, we initially assessed SIRT1 protein expression and overall protein acetylation levels after NMN treatment. Western blotting revealed that NMN treatment significantly increased SIRT1 protein expression in FGSCs (Fig. 2A) while markedly reducing acetylation levels of histone regions (Fig. 2B). To identify proteins undergoing acetylation changes post-NMN treatment, we performed quantitative acetylation profiling on FGSC samples collected before and after NMN treatment. This analysis identified 578 differentially expressed acetylated proteins (DEAPs) and 686 differentially expressed acetylated sites (DEASs) between the control and NMN-treated groups (Fig. 2C, D). Subcellular localization analysis showed that DEAPs were predominantly found in the cytoplasm (42.67%), with substantial distributions in the nucleus (27.83%) and mitochondria (10.2%) (Fig. 2E).

To elucidate the potential mechanisms by which NMN-induced acetylation affects FGSC development, we conducted Gene Ontology (GO) and Kyoto Encyclopedia of Genes and Genomes (KEGG) enrichment analyses. GO enrichment analysis revealed that DEAPs were mainly involved in biological processes such as chromatin binding, cell cycle, histone acetylation, cell population proliferation, and H4 histone acetyltransferase activity (Fig. 2F). KEGG pathway analysis indicated that DEAPs participated in pathways including RNA transport, DNA replication, cell cycle, regulation of actin cytoskeleton, and the HIF-1 signaling pathway (Fig. 2G).

Further GO enrichment analysis linked NMN treatment in FGSCs to biological processes associated with histone acetylation and H4 histone acetyltransferase activity. We identified 18 differentially acetylated histone modification sites, with 13 located in nucleosome core histone subunits. Among these, eight sites showed increased acetylation (H4K16, H2K23, H4K12, H4K8, H2K16, H2K20, H3K14, H4K91), while five showed decreased acetylation (H2K16, H3K27, H3K36, H2K116, H4K79) (Fig. 2H). Notably, H4K16 showed the highest fold change among upregulated sites, indicating its critical role in NMN-induced FGSC development (Fig. 2H). Western blotting confirmed that NMN treatment significantly increased H4K16 acetylation levels in FGSCs (Fig. 2I), and secondary mass spectrometry further verified H4K16 acetylation (Fig. 2J).

Next, we examined whether NMN’s enhancement of FGSC viability and proliferation is mediated through H4K16 acetylation (H4K16ac). Western blotting indicated that MC4033 treatment significantly reduced H4K16ac levels, with the strongest effect at 100 μM (Fig. S1). Co-treatment with MC4033 also significantly attenuated NMN’s enhancing effect on H4K16ac levels (Fig. 2K). CCK-8 assays showed that MC4033 alone significantly reduced FGSC viability, and co-treatment diminished NMN’s enhancement of cell viability (Fig. 2L). EdU assays demonstrated that MC4033 treatment significantly decreased FGSC proliferation, and co-treatment weakened NMN’s promotive effect on cell proliferation (Fig. 2M). These findings demonstrate that NMN treatment significantly promotes H4K16ac in FGSCs, thereby enhancing cell viability and proliferation.

### NMN enhances FGSC viability and proliferation by upregulating *Hmgb1* expression through histone H4K16ac modification

**Fig. 3:**
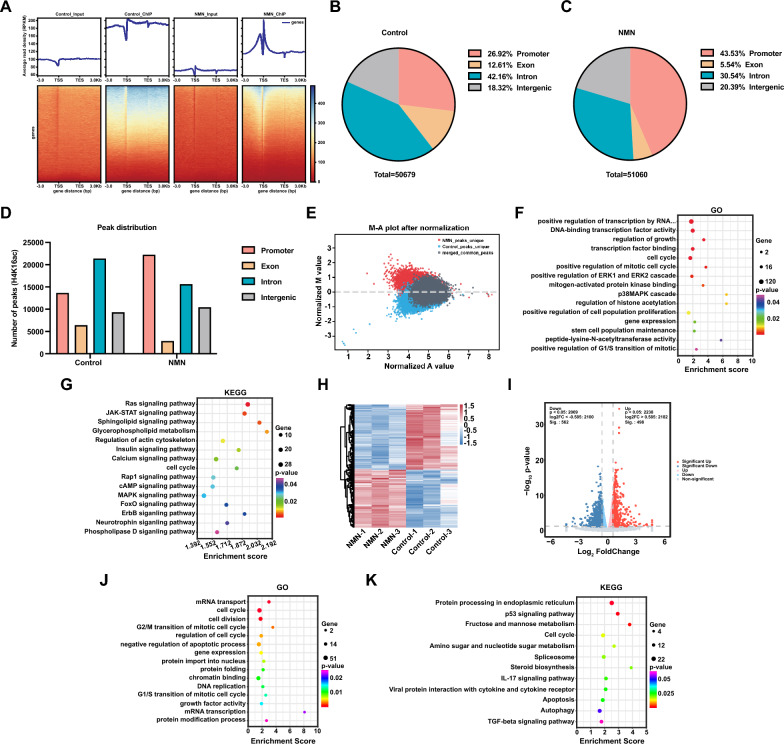
ChIP-seq and RNA-seq analyses in control and NMN-treated groups. **A** Heatmap displaying gene body enrichment from ChIP-seq data. Peak distribution in control (**B**) and NMN-treated (**C**) groups. **D** Statistical comparison of peak distributions between control and NMN groups. **E** MA plot showing differentially expressed peaks between the groups. GO (**F**) and KEGG pathway (**G**) enrichment analyses for genes associated with differential peaks. Hierarchical clustering heatmap (**H**), volcano plot (**I**), and GO (**J**) and KEGG pathway (**K**) enrichment analyses of DEGs between the groups

To elucidate the mechanisms by which increased H4K16ac modification  affects FGSC development, we collected FGSC samples before and after NMN treatment for ChIP-seq and RNA-seq analyses. The gene body enrichment heatmap showed significant enrichment of ChIP data near the transcription start site (TSS) compared to Input. Notably, the NMN-treated group exhibited higher enrichment in the TSS region than the control group (Fig. 3A). Peak distribution analysis indicated that in both groups, peaks were primarily enriched in intronic (42.16% and 30.54%) and promoter (26.92% and 43.53%) regions, with smaller fractions in exonic (12.61% and 5.54%) and intergenic (18.32% and 20.39%) regions (Fig. 3B, C). The number of peaks enriched in the promoter region increased approximately 1.6-fold in the NMN-treated group compared to the control (Fig. 3D). The MA-value plot revealed that 41,515 peaks were uniquely enriched in the control group, while 39,362 peaks were exclusively enriched in the NMN-treated group (Fig. 3E).

GO enrichment analysis revealed that differentially expressed genes (DEGs) were primarily involved in biological processes such as regulation of growth, transcription factor binding, cell cycle, regulation of histone acetylation, positive regulation of cell population proliferation, gene expression, and stem cell populations maintenance (Fig. 3F). KEGG pathway analysis indicated that DEGs were associated with signaling pathways, including Ras, JAK-STAT, cell cycle, Rap1, MAPK, and Phospholipase D signaling pathways (Fig. 3G).

RNA-seq results showed that after NMN treatment, 498 genes were significantly upregulated, while 562 genes were significantly downregulated (Fig. 3H, I). GO enrichment analysis indicated that DEGs were involved in biological processes like mRNA transport, cell cycle, cell division, negative regulation of apoptotic process, gene expression, and chromatin binding (Fig. 3J). KEGG pathway analysis revealed associations with pathways such as the p53 signaling pathway, cell cycle, apoptosis, autophagy, and the TGF-β signaling pathway (Fig. 3K).

**Fig. 4:**
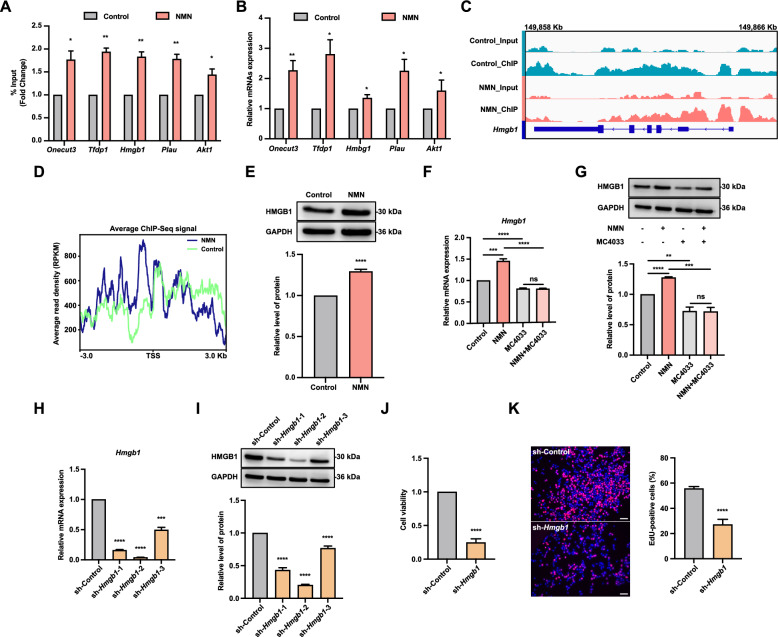
NMN enhances FGSC viability and proliferation by upregulating *Hmgb1* expression through histone H4K16ac modification. **A** ChIP-qPCR analysis of *Hmgb1*, *Onecut3*, *Tfdp1*, *Akt1*, and *Plau* gene enrichment levels in control and NMN-treated groups. **B** qRT-PCR analysis of *Hmgb1*, *Onecut3*, *Tfdp1*, *Akt1*, and *Plau* gene expression levels in control and NMN-treated groups. **C** IGV visualization of H4K16ac enrichment in the *Hmgb1* gene. **D** Enrichment of H4K16ac modifications within ±3 Kb of the *Hmgb1* gene’s TSS. **E** NMN treatment increases HMGB1 protein expression. **F** qRT-PCR analysis of *Hmgb1* expression after combined treatment with MC4033. **G** Western blot analysis of HMGB1 protein levels following MC4033 co-treatment. Knockdown efficiency of various lentiviral constructs targeting *Hmgb1* in FGSCs assessed by qRT-PCR (**H**) and western blotting (**I**). **J** Cell viability assay comparing control and *Hmgb1*-knockdown groups. **K** Representative EdU staining images and the proportion of EdU-positive cells in control and *Hmgb1* knockdown groups. * *p* < 0.05, ** *p* < 0.01, *** *p* < 0.001, **** *p* < 0.0001, ^ns^ *p* > 0.05. Scale bars: 50 μm

To validate ChIP-seq and RNA-seq results, we selected five genes (*Onecut3*, *Tfdp1*, *Hmgb1*, *Plau*, and *Akt1*) that exhibited increased H4K16ac enrichment and upregulation in expression following NMN treatment for ChIP-qPCR and qRT-PCR analysis. Results indicated that NMN treatment significantly increased H4K16ac enrichment at the *Onecut3*, *Tfdp1*, *Hmgb1*, *Plau*, and *Akt1* genes compared to the control group (Fig. 4A), and also elevated the expression levels of these genes (Fig. 4B). Integrative Genomics Viewer (IGV) visualization indicated stronger signal enrichment at the promoter region of the *Hmgb1* gene in the NMN-treated group (Fig. 4C). Further ChIP-seq analysis revealed higher enrichment signals in the region 3 Kb upstream of the *Hmgb1* TSS in the NMN-treated group compared to the control (Fig. 4D). These results identified *Hmgb1* as a potential target gene of the H4K16ac protein. Western blotting confirmed that NMN treatment significantly enhanced HMGB1 protein expression in FGSCs (Fig. 4E). Additionally, qRT-PCR indicated that MC4033 treatment alone significantly reduced *Hmgb1* expression, and co-treatment with MC4033 significantly diminished NMN’s effect on *Hmgb1* expression (Fig. 4F). Correspondingly, western blotting showed that MC4033 treatment alone significantly decreased HMGB1 protein levels, and co-treatment markedly attenuated NMN’s enhancing effect (Fig. 4G). These findings indicate that NMN upregulates *Hmgb1* expression through histone H4K16 acetylation modification.

To investigate the role of *Hmgb1* in FGSC development, we performed lentiviral infection of FGSCs using a knockdown control and three different *Hmgb1* knockdown lentiviruses. qRT-PCR and western blotting indicated that all three lentiviruses significantly reduced *Hmgb1* expression compared to the knockdown control group (Fig. 4H, I). CCK-8 assays showed that *Hmgb1* knockdown significantly reduced FGSC viability compared to the control (Fig. 4J). Furthermore, EdU assay demonstrated that *Hmgb1* knockdown significantly decreased FGSC proliferative capacity comparison to the control (Fig. 4K).

We further investigated whether NMN’s enhancement of FGSC viability and proliferation is mediated through *Hmgb1*. Results from qRT-PCR and western blotting indicated no significant change in *Hmgb1* expression in the NMN co-treatment group compared to the *Hmgb1* knockdown group (Fig. S2A, B). Additionally, CCK-8 and EdU assays showed no significant changes in cell viability and proliferation capacity in the NMN co-treatment group compared to the *Hmgb1* knockdown group (Fig. S2C, D). These findings indicate that NMN enhances FGSC viability and proliferation by upregulating *Hmgb1* expression through histone H4K16 acetylation modification.

### *Hmgb1* knockdown inhibits FGSC cell cycle progression and promotes apoptosis

**Fig. 5:**
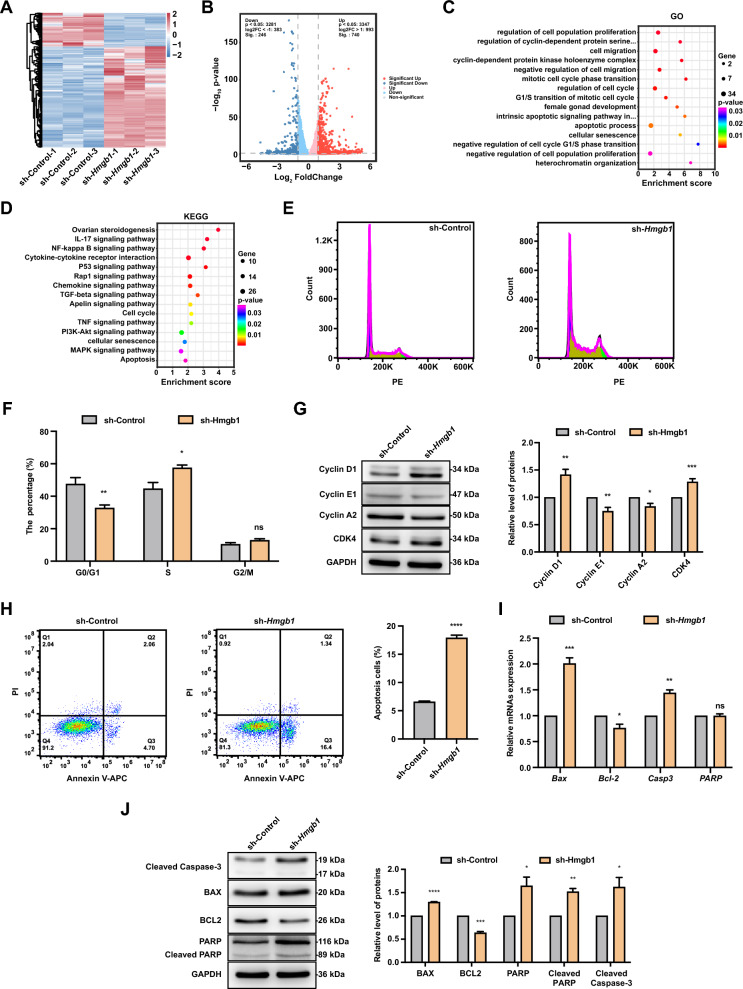
*Hmgb1* knockdown inhibits FGSC cell cycle progression and promotes apoptosis. Hierarchical clustering heatmap (**A**) and volcano plot (**B**) showing DEGs between control and *Hmgb1* knockdown groups. GO (**C**) and KEGG pathway (**D**) enrichment analyses of DEGs between the two groups. **E** Flow cytometry analysis of cell cycle distribution in control and *Hmgb1* knockdown groups. **F** Statistical comparison of cell populations across different cell cycle phases for both groups. **G** Western blot analysis of cyclin D1, cyclin E1, cyclin A2, and CDK4 expression levels in control and *Hmgb1* knockdown groups. **H** Flow cytometry analysis of apoptosis in control and *Hmgb1* knockdown groups. **I** Impact of *Hmgb1* knockdown on the expression of apoptosis-related genes. **J** Western blot analysis of cleaved-Caspase3, BAX, BCL2, PARP, and cleaved-PARP levels in control and *Hmgb1* knockdown groups. * *p* < 0.05, ** *p* < 0.01, *** *p* < 0.001, **** *p* < 0.0001, ^ns^ *p* > 0.05

To elucidate the mechanisms by which increased *Hmgb1* expression affects FGSC development, we collected FGSC samples before and after *Hmgb1* knockdown for RNA-seq analysis. Results showed that *Hmgb1* knockdown led to significant changes in gene expression, with 740 genes upregulated and 246 downregulated (Fig. 5A, B). GO enrichment analysis indicated that the DEGs were mainly involved in processes such as regulation of cell population proliferation, cell migration, regulation of cell cycle, apoptotic processes, cellular senescence, and heterochromatin organization (Fig. 5C). KEGG pathway analysis linked the DEGs to signaling pathways including NF-kappa B, Rap1, TGF-β, cell cycle, PI3K-Akt, cellular senescence, MAPK, and apoptosis (Fig. 5D).

Further analysis suggested that *Hmgb1* knockdown was associated with biological processes related to cell cycle regulation and apoptosis. Flow cytometry revealed that *Hmgb1* knockdown significantly decreased the proportion of cells in the G0/G1 phase while increasing the S phase population, with no significant changes in the G2/M phase (Fig. 5E, F). Western blotting showed that *Hmgb1* knockdown significantly upregulated Cyclin D1 and CDK4 protein levels, while Cyclin E2 and Cyclin A2 levels were significantly downregulated compared to the control group (Fig. 5G). These findings suggest that *Hmgb1* knockdown may inhibit FGSC cell cycle progression.

Apoptosis analysis by flow cytometry demonstrated that *Hmgb1* knockdown significantly increased the percentage of apoptotic cells, from 6.76% in the control to 17.74%, primarily in the early apoptosis stage (Fig. 5H). qRT-PCR results demonstrated that *Hmgb1* knockdown significantly elevated the expression of *Bax* and *Casp3*, while reducing *Bcl-2* expression (Fig. 5I). Western blotting confirmed increased levels of BAX, PARP, cleaved PARP, and cleaved Caspase-3, alongside decreased BCL2 following *Hmgb1* knockdown (Fig. 5J). These findings indicate that *Hmgb1* knockdown inhibits FGSC proliferation by disrupting cell cycle regulation and promoting apoptosis.

### *Hmgb1* knockdown affects chromatin accessibility and alters the higher-order chromatin structure in FGSCs

GO enrichment analysis of DEGs identified biological processes associated with heterochromatin assembly and chromatin accessibility following *Hmgb1* knockdown (Fig. 5C). These findings suggest that *Hmgb1* knockdown may influence chromatin accessibility and higher-order chromatin structure in FGSCs. To further investigate, we performed ATAC-seq and Hi-C analyses on FGSC samples collected before and after *Hmgb1* knockdown treatment. The TSS enrichment heatmap showed that ATAC-seq data from the *Hmgb1* knockdown group exhibited reduced enrichment at TSS regions compared to the control group (Fig. S3A). Peaks distribution analysis revealed that, in the control group, 15,287 peaks (27.28%) were enriched in promoter regions, 1979 peaks (3.53%) in exon regions, 16,700 peaks (29.8%) in intron regions, and 22,069 peaks (39.38%) in distal intergenic regions (Fig. S3B, C). In contrast, the *Hmgb1* knockdown group showed 13,477 peaks (33.28%) enriched in promoter regions, 1434 peaks (3.54%) in exon regions, 10,783 peaks (26.62%) in intron regions, and 14,806 peaks (36.56%) in distal intergenic regions (Fig. S3B, C). The volcano plot of differentially enriched peaks revealed 136 peaks were highly enriched in the *Hmgb1* knockdown group, while 25,542 peaks were highly enriched in the control group (Fig. S3D).

GO enrichment analysis revealed that the DEGs were primarily involved in biological processes such as apoptosis, negative regulation of cell population proliferation, chromatin binding, stem cell proliferation, chromatin remodeling, chromatin assembly, and cell cycle regulation (Fig. S3E). KEGG pathway enrichment analysis further indicated associations with signaling pathways including PI3K-Akt, Rap1, MAPK, apoptosis, phospholipase D, NF-kappa B, and cell cycle pathways (Fig. S3F).

**Fig. 6:**
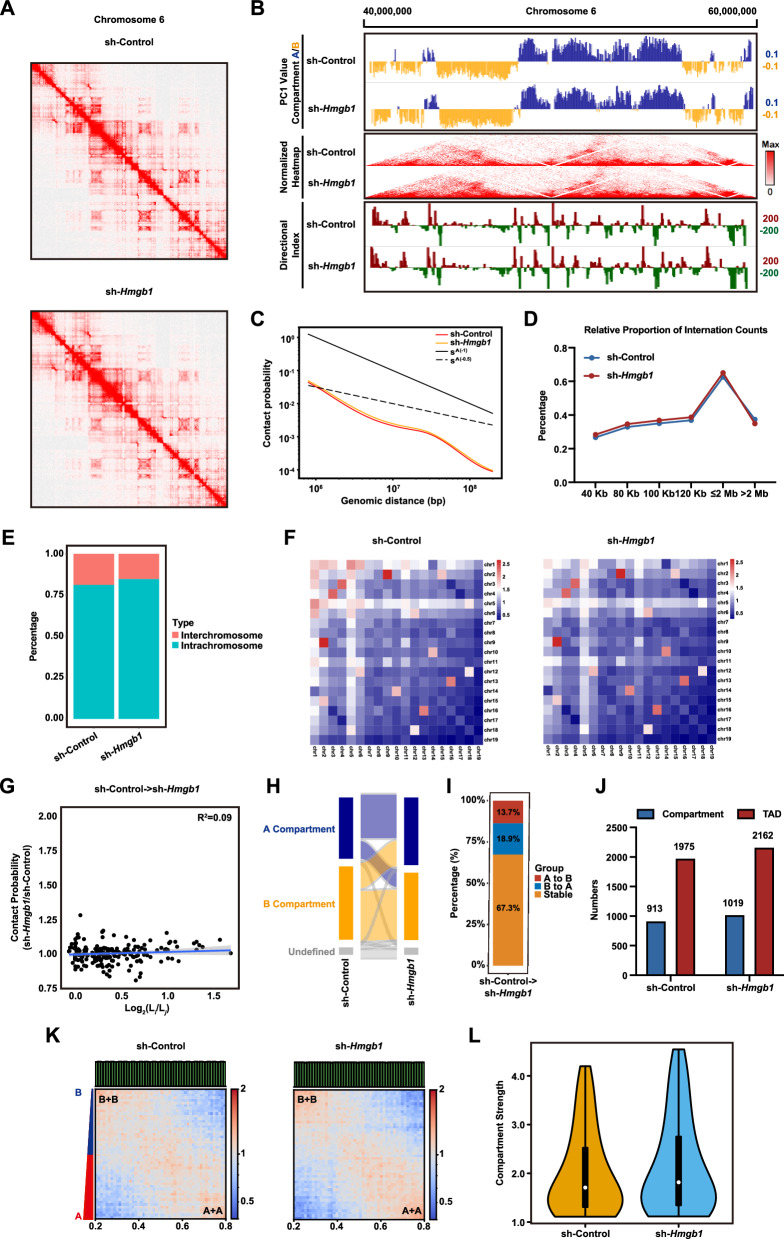
Effects of *Hmgb1* knockdown on the higher-order chromatin structure in FGSCs. **A** Normalized Hi-C interaction matrix heatmap. **B** Normalized Hi-C interaction heatmap at 20 Kb resolution, directional index (DI), and the first principal component (PC1) values. PC1 values represent A/B compartments, with positive PC1 values (blue) indicating compartment A and negative PC1 values (yellow) indicating compartment B. **C** Decay curve of the average contact probability across chromosomes. **D** Relative proportions of total cis interactions for different genomic distances and total paired loci. **E** Percentages of intra-chromosomal and inter-chromosomal contacts before and after knockdown. **F** Observed/Expected (O/E) contact numbers between any pair of chromosomes before and after knockdown. **G** O/E numbers of inter-chromosomal interactions normalized by chromosome length for autosomes. **H** Sankey diagram showing dynamic changes in A/B compartment states before and after *Hmgb1* knockdown. **I** Percentage distribution of A/B compartments that underwent state transitions in the genome before and after *Hmgb1* knockdown. **J** Statistical comparison of A/B compartments and TAD numbers. **K** Saddle plot depicting the compartmentalization strength of (A + A) and (B + B) interactions before and after *Hmgb1* knockdown. **L** Violin plot showing changes in compartmentalization strength before and after *Hmgb1* knockdown

Next, we generated high-resolution chromatin architecture maps of FGSC development before and after *Hmgb1* knockdown. The results revealed that *Hmgb1* knockdown altered the spatial chromatin structure of FGSCs (Fig. 6A). Compared to the control group, the chromatin interaction heatmap of chromosome 6 exhibited increased “triangular” structures and elevated directional index (DI) values, indicating a stronger preference for upstream and downstream interactions. Additionally, inter-compartmental transitions were observed following *Hmgb1* knockdown (Fig. 6B). Average interaction probability decay curves showed similar power-law exponents between the knockdown and control groups, with chromatin contact probabilities gradually decreasing as genomic distance increased (Fig. 6C). Furthermore, short-range (≤2 Mb) intra-chromosomal interactions were more frequent in the *Hmgb1* knockdown group, while long-range interactions (>2 Mb) were proportionally higher in the control group (Fig. 6D).

Next, analysis of long-range intra- and inter-chromosomal interactions revealed that intra-chromosomal interactions were more frequent than inter-chromosomal interactions. After *Hmgb1* knockdown, the proportion of intra-chromosomal interactions increased, while inter-chromosomal interactions decreased compared to the control group (Fig. 6E). Observed/expected (O/E) contact analysis among autosomes showed a declining trend in inter-chromosomal interaction frequency following *Hmgb1* knockdown, with chromosome 1 displaying stronger interactions with other chromosomes (Fig. 6F). Statistical analysis of inter-chromosomal contact probabilities indicated no significant differences in interaction frequencies between long and short chromosomes before and after the knockdown treatment (Fig. 6G).

We then examined the dynamic changes in chromatin spatial organization at the A/B compartment level following *Hmgb1* knockdown. The results revealed A/B compartment switching, characterized by an increased proportion of A compartments and a corresponding decrease in B compartments (Fig. 6H). Notably, 32.6% of genomic regions underwent A/B compartment transitions after *Hmgb1* knockdown (Fig. 6I). The total number of A/B compartments also increased, with 913 compartments identified in the control group and 1019 in the *Hmgb1* knockdown group (Fig. 6J). Eigenvector analysis demonstrated that both groups exhibited strong intra-compartment interactions and weaker inter-compartment interactions (Fig. 6K). Additionally, no significant difference in compartmentalization strength was observed between the two groups (Fig. 6L).

We next analyzed the dynamic changes in chromatin spatial organization at the TAD (topologically associating domain) level following *Hmgb1* knockdown. The number of TADs increased from 1975 in the control group to 2162 in the *Hmgb1* knockdown group (Fig. 6J). Genome-wide analysis revealed no significant differences in DI values between the two groups (Fig. S4A). Insulation score analysis, which quantifies TAD strength, showed high variability in insulation score curves for both groups but no significant differences (Fig. S4B). Resampling observed reads as observed/expected (O/E) to assess intra-TAD interaction frequencies revealed values of 1.45 for the control group and 1.46 for the *Hmgb1* knockdown group (Fig. S4C). TAD boundary score analysis further confirmed significantly higher boundary scores in the knockdown group, reflecting strengthened TAD boundaries and reduced interactions between adjacent TADs (Fig. S4D). Additionally, intra-TAD interactions were significantly increased following *Hmgb1* knockdown (Fig. S4E).

We further analyzed the dynamic changes in chromatin spatial organization at the chromatin loop level following *Hmgb1* knockdown. The results revealed a reduction in the number of chromatin loops, with 7281 loops identified in the control group compared to 7207 in the *Hmgb1* knockdown group (Fig. S4F). *Hmgb1* knockdown also decreased the interaction frequency between gene fragments within chromatin loops (Fig. S4G). Statistical analysis confirmed that the interaction frequency within chromatin loops was significantly lower in the knockdown group than in the control group (Fig. S4H). These findings indicate that *Hmgb1* knockdown impacts chromatin accessibility and alters the higher-order chromatin structure in FGSCs.

### *Fyn* overexpression reverses the inhibition of FGSC proliferation induced by *Hmgb1* knockdown

**Fig. 7:**
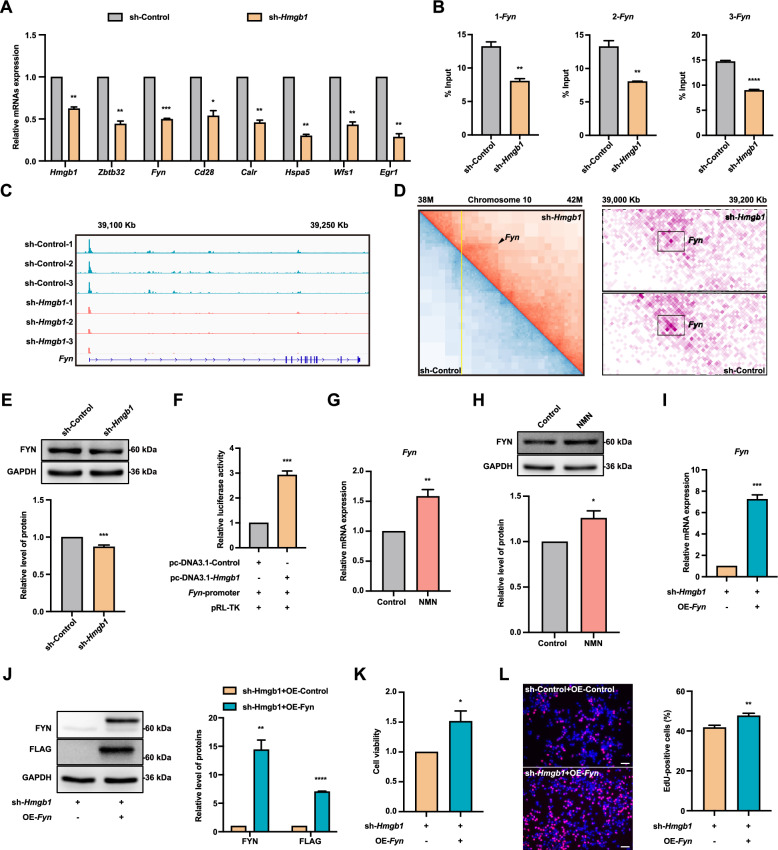
*Fyn* overexpression reverses the inhibition of FGSC proliferation induced by *Hmgb1* knockdown. A qRT-PCR analysis of gene expression for *Hmgb1*, *Zbtb32*, *Fyn*, *Cd28*, *Calr*, *Hspa5*, *Wfs1*, and *Egr1* in control and *Hmgb1* knockdown groups. **B** ChIP-qPCR analysis showing *Fyn* gene enrichment in control and *Hmgb1* knockdown groups. **C** IGV visualization analysis of HMGB1 enrichment in the *Fyn* gene. **D** Heatmap showing the changes in chromatin loops in the *Fyn* promoter region before and after *Hmgb1* knockdown. **E** *Hmgb1* knockdown reduces FYN protein expression. **F** Dual-luciferase reporter assay demonstrating the interaction between the *Fyn* gene promoter region and *Hmgb1*. Assessment of *Fyn* expression levels in control and NMN-treated groups using qRT-PCR (**G**) and western blotting (**H**). Evaluation of *Fyn* overexpression effects via qRT-PCR (**I**) and western blotting (**J**). **K** Cell viability assay following *Fyn* overexpression. **L** Representative images of EdU staining and the percentage of EdU-positive cells after *Fyn* overexpression. * *p* < 0.05, ** *p* < 0.01, *** *p* < 0.001, **** *p* < 0.0001. Scale bars: 50 μm

To explore the target genes of *Hmgb1* and their role in FGSC development, seven genes (*Hmgb1*, *Zbtb32*, *Fyn*, *Cd28*, *Calr*, *Hspa5*, *Wfs1*, and *Egr1*) that showed reduced expression after *Hmgb1* knockdown in RNA-seq analysis were selected for qRT-PCR validation. The qRT-PCR results confirmed that all seven genes were significantly downregulated, consistent with the RNA-seq results (Fig. 7A). Notably, *Fyn* showed the most significant decrease in expression, suggesting that it may play an important role as a target gene of *Hmgb1*. ChIP-qPCR analysis using three primer pairs for the *Fyn* promoter region showed reduced HMGB1 enrichment at *Fyn* in the *Hmgb1* knockdown group (Fig. 7B). IGV visualization revealed stronger signal enrichment at the promoter region of the *Fyn* gene in the control group (Fig. 7C). Moreover, Hi-C data demonstrated that the chromatin loop structure at the *Fyn* promoter region disappeared after *Hmgb1* knockdown (Fig. 7D). Based on these findings, we identified *Fyn* as a potential target gene of *Hmgb1*. Western blotting further confirmed that FYN protein levels were significantly decreased after *Hmgb1* knockdown (Fig. 7E). Additionally, a dual-luciferase reporter assay demonstrated that HMGB1 overexpression significantly enhanced luciferase activity, supporting the interaction between *Hmgb1* and the *Fyn* promoter (Fig. 7F). NMN treatment significantly increase *Fyn* transcription and protein expression in FGSCs (Fig. 7G, H). Results from qRT-PCR and western blotting indicated no significant change in *Fyn* expression in the NMN co-treatment group compared to the *Hmgb1* knockdown group (Fig. S2E, F).

To determine if *Fyn* overexpression could counteract the effects of *Hmgb1* knockdown, *Hmgb1* knockdown FGSCs were infected with either a control or *Fyn* overexpression lentivirus. The qRT-PCR and western blotting results confirmed that *Fyn* expression was significantly elevated in the *Fyn* overexpression group compared to controls (Fig. 7I, J). CCK-8 and EdU assays showed that *Fyn* overexpression significantly reversed the suppression of cell viability and proliferation caused by *Hmgb1* knockdown (Fig. 7K, L). These findings indicate that *Fyn* overexpression reverses the inhibitory effect of *Hmgb1* knockdown on FGSC proliferation.

### *Fyn* overexpression enhances FGSC viability and proliferation via the phospholipase D signaling pathway

**Fig. 8:**
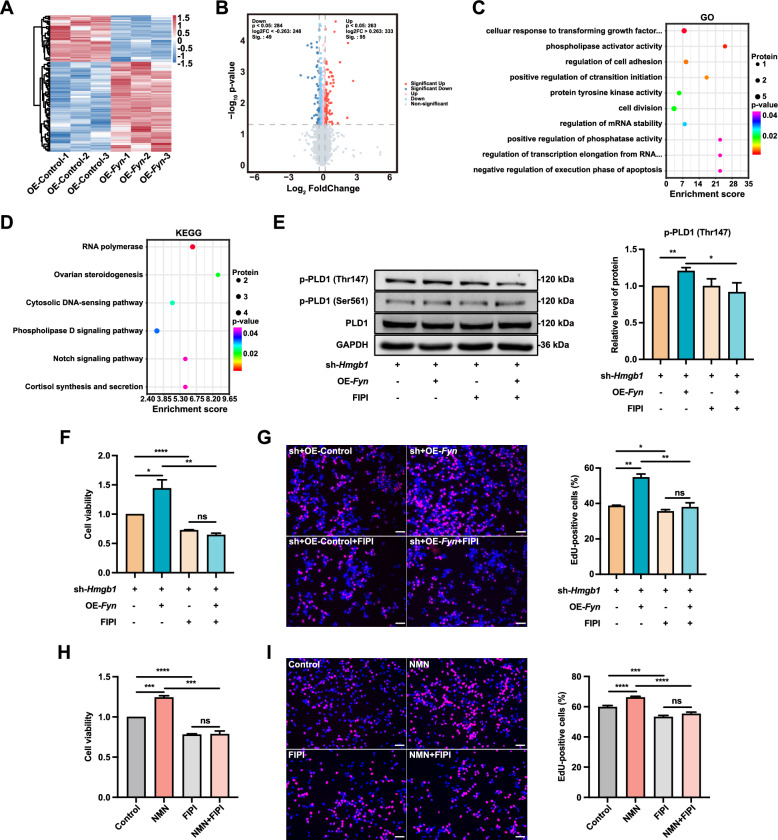
*Fyn* overexpression enhances FGSC viability and proliferation via the phospholipase D signaling pathway. Hierarchical clustering heatmap (**A**), volcano plot (**B**), and GO (**C**) and KEGG pathway (**D**) enrichment analyses of DEPs between control and *Fyn* overexpression groups. **E** Co-treatment with FIPI reverses the *Fyn* overexpression-induced increase in PLD1 phosphorylation at Thr147. **F** FIPI co-treatment decreases the *Fyn* overexpression-induced enhancement of FGSC viability. **G** FIPI co-treatment reduces the *Fyn* overexpression-induced enhancement of FGSC proliferation. **H** FIPI co-treatment attenuates the NMN-induced increase in FGSC viability. **I** FIPI co-treatment diminishes the NMN-induced enhancement of FGSC proliferation. * *p* < 0.05, ** *p* < 0.01, *** *p* < 0.001, **** *p* < 0.0001, ^ns^ *p* > 0.05. Scale bars: 50 μm

To understand how *Fyn* overexpression counteracts the effects of *Hmgb1* knockdown on FGSC development, we conducted proteomic analysis of FGSCs before and after *Fyn* overexpression. The results showed that 95 proteins were significantly upregulated and 49 were downregulated (Fig. 8A, B). GO enrichment analysis indicated that the differentially expressed proteins (DEPs) were primarily associated with biological processes such as phospholipase activator activity, regulation of cell adhesion, protein tyrosine kinase activity, cell division, and regulation of mRNA stability (Fig. 8C). KEGG pathway analysis identified the DEPs as being linked mainly to RNA polymerase, ovarian steroidogenesis, and the phospholipase D signaling pathway (Fig. 8D).

The identification of the phospholipase D (PLD) signaling pathway suggested its key role in reversing the effects of *Hmgb1* knockdown. Western blot analysis revealed that *Fyn* overexpression significantly increased PLD1 phosphorylation at Thr147, but not at Serine 561 (Ser561), compared to controls. This effect was reversed by FIPI co-treatment (Fig. 8E). To assess whether FIPI affected the Fyn-induced improvements in FGSC viability and proliferation, CCK-8 and EdU assays showed that FIPI treatment alone significantly reduced cell viability and proliferation. Additionally, co-treatment with FIPI markedly diminished the positive effects of Fyn overexpression on these parameters (Fig. 8F, G).

We then tested whether FIPI treatment could influence the NMN-induced enhancement of FGSC viability and proliferation. CCK-8 and EdU assays showed that FIPI alone significantly reduced FGSC viability and proliferation. Moreover, combining FIPI with NMN markedly weakened NMN’s positive effects on these parameters (Fig. 8H, I). These findings indicate that *Fyn* overexpression enhances FGSC viability and proliferation through the PLD signaling pathway.

## Discussion

FGSCs offer a promising approach for understanding the mechanisms of female infertility and exploring potential treatments, with enhancing their proliferation efficiency being crucial for practical applications. In this study, we demonstrated that NMN significantly enhanced FGSC viability and proliferation while also increasing  the modification level  of H4K16ac. ChIP-seq and RNA-seq identified *Hmgb1* as a downstream target of H4K16ac. *Hmgb1* knockdown inhibited FGSC proliferation by disrupting cell cycle progression, inducing apoptosis, reducing chromatin accessibility, and altering 3D chromatin structure. Further analyses using ATAC-seq, Hi-C, and RNA-seq revealed that *Fyn* acts as a downstream target of *Hmgb1*. *Fyn* overexpression restored the FGSC proliferation that had been suppressed by *Hmgb1* knockdown. This rescue effect was mediated through the PLD signaling pathway, specifically by enhancing the phosphorylation of PLD1 at threonine 147. The pro-proliferative effect of *Fyn* overexpression was blocked by FIPI.

NMN, a nucleotide, primarily serves as a precursor for NAD^+^ production [39]. SIRT1, an NAD^+^-dependent histone deacetylase, targets multiple lysine residues for deacetylation, including lysine 4, 12, and 16 on histone H4, as well as lysine 9, 14, 18, and 56 on histone H3 [40–42]. In this study, NMN significantly upregulated SIRT1 expression in FGSCs. Interestingly, while NMN reduced overall histone acetylation levels in FGSCs, specific acetylation sites, particularly on histone H4 (lysines 16, 12, 8, and 91) and histone H3 (lysine 14), showed significant increases. This discrepancy may be attributed to the influence of the NAD^+^/NADH ratio on SIRT1 activity [43, 44]. H4K16ac, a recognized marker of transcriptional and enhancer activity [40, 45, 46], is primarily associated with processes such as cell cycle regulation, DNA repair, replication, autophagy, and differentiation [47–53]. Among the upregulated acetylation sites, lysine 16 on histone H4 showed the most pronounced change, suggesting its critical role in NMN-mediated enhancement of FGSC proliferation.

*Hmgb1*, a member of the high mobility group superfamily, is located in the nucleus and cytoplasm and can also be secreted extracellularly [54]. The present results indicated that NMN regulates cell viability and proliferation by modifying H4K16ac on *Hmgb1*. Previous studies have shown that *Hmgb1* is involved in various cellular processes, including cell proliferation, differentiation, inflammation, immune response, migration, and tissue regeneration [55–64]. In this study, *Hmgb1* knockdown significantly reduced FGSC viability and proliferation. GO analysis revealed enrichment in pathways related to cell migration, cell cycle regulation, G1/S transition of the mitotic cell cycle, and apoptosis. Western blotting and flow cytometry analysis confirmed that *Hmgb1* knockdown influences cell cycle progression and apoptotic pathways. Additionally, *Hmgb1* knockdown was associated with the enrichment of several signaling pathways, including TGF-β, TNF, PI3K-Akt, and MAPK signaling pathways. These findings suggest that *Hmgb1* may indirectly regulate FGSC proliferation through these signaling pathways.

As a non-histone transcription factor, *Hmgb1* regulates gene expression to control cell proliferation [65]. In this study, *Fyn* was identified as a novel target gene regulated by *Hmgb1* in FGSCs. Silencing *Hmgb1* led to a reduction in both *Fyn* mRNA and protein levels. *Fyn*, a member of the Src family, is known to participate in cell migration, proliferation, and metastasis via the PI3K/Akt and MAPK/ERK signaling pathways [66–68]. *Fyn* overexpression significantly reversed the inhibitory effects of *Hmgb1* knockdown on cell viability and proliferation. Proteome sequencing in *Fyn* overexpressing cells further clarified the mechanisms by which increased *Fyn* levels counteracted the inhibition of FGSC proliferation caused by *Hmgb1* knockdown. Compared with FGSCs with *Hmgb1* knockdown alone, *Fyn* overexpressing cells showed enrichment in the PLD signaling pathway, suggesting that *Fyn* may regulate cell viability and proliferation in FGSCs through this pathway. The PLD signaling pathway plays a crucial role in various biological processes, including cell differentiation, proliferation, motility, and the endoplasmic reticulum stress response [69–71]. In this study, *Fyn* overexpression significantly increased the phosphorylation of PLD1 at Thr147. Moreover, treatment with FIPI counteracted the pro-proliferative effects of *Fyn* overexpression on FGSCs. Together, these findings highlight the pivotal role of the PLD signaling pathway in FGSCs, functioning downstream of *Fyn* to regulate cell viability and proliferation.

## Conclusions

**Fig. 9:**
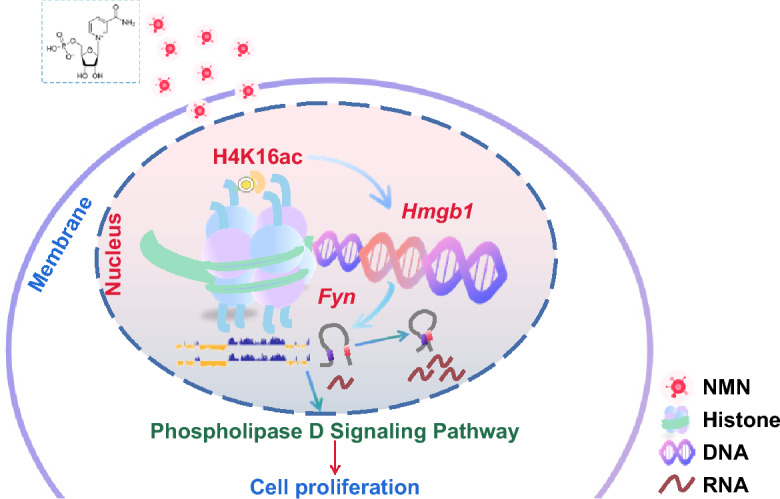
Schematic diagram illustrating NMN promotes FGSC proliferation. NMN promotes FGSC proliferation by activating the H4K16ac-*Hmgb1*-*Fyn*-PLD signaling pathway through epigenetic remodeling

In conclusion, this study demonstrated that NMN promotes FGSC proliferation by activating the H4K16ac-*Hmgb1*-*Fyn*-PLD signaling pathway through epigenetic remodeling, as illustrated in Fig. 9. These findings lay a foundation for a deeper understanding of the regulatory mechanisms driving FGSC proliferation and open up new avenues for the practical application of FGSCs in reproductive medicine.

## Supplementary Information

Additional file 1 includes Figures S1 to S4 and Tables S1 to S5.

Additional file 2 contains the full uncropped blot images.

## Data Availability

The ChIP-seq, RNA-seq, ATAC-seq, and Hi-C data supporting the findings of this study are available in the NCBI Gene Expression Omnibus database under accession number GSE248653, GSE286934, and GSE286936. The mass spectrometry proteomics data have been submitted to the ProteomeXchange Consortium and can be accessed via dataset identifiers PXD047214 and PXD047216.

## Abbreviations

NMN: Nicotinamide mononucleotide
NAD^+^: Nicotinamide adenine dinucleotide
SIRT1: Sirtuin1
FGSCs: Female germline stem cells
MAPK: Mitogen-activated protein kinase
PI3K: Phosphatidylinositol-3-kinase
Wnt: Wingless/integrated
H4K16ac: Histone H4 lysine 16 acetylation
ChIP-seq: Chromatin immunoprecipitation sequencing
RNA-seq: RNA-sequencing
*Hmgb1*: High mobility group box 1
ATAC-seq: Assay for transposase-accessible chromatin sequencing
Hi-C: High-throughput chromosome conformation capture
*Fyn*: Fyn proto-oncogene
PLD: Phospholipase D
Thr147: Threonine 147
FIPI: 5-Fluoro-2-indolyldechlorohaloamide
PBS: Phosphate-buffered saline
DMSO: Dimethyl sulfoxide
HBSS: Hank’s balanced salt solution
STO: SIM mouse embryo-derived thioguanine- and ouabain-resistant
CCK-8: Cell counting kit-8
EdU: 5-Ethynyl-2′-deoxyuridine
qRT-PCR: Quantitative real-time PCR
MOI: Multiplicity of infection
DEAPs: Differentially expressed acetylation-modified proteins
DEASs: Differentially expressed acetylation-modified sites
GO: Gene ontology
KEGG: Kyoto encyclopedia of genes and genomes
TSS: Transcription start site
DEGs: Differentially expressed genes
IGV: Integrative genomics viewer
DI: Directional index
O/E: Observed/expected
TAD: Topologically associating domain
DEPs: Differentially expressed proteins
Ser561: Serine 561
