# Supplementary material for: Nicotinamide mononucleotide promotes female germline stem cell proliferation by activating the H4K16ac-Hmgb1-Fyn-PLD signaling pathway through epigenetic remodeling
Source: Cell Biosci. 2025 Apr 17;15:48. doi: 10.1186/s13578-025-01387-w (PMC12004683; doi:10.1186/s13578-025-01387-w)
Supplement: Supplementary file 1 — Additional file 1 includes Figures S1 to S4 and Tables S1 to S5. [file 13578_2025_1387_MOESM1_ESM.docx]

**Nicotinamide Mononucleotide Promotes Female Germline Stem Cell Proliferation by Activating the H4K16ac-*Hmgb1*-*Fyn*-PLD Signaling Pathway through Epigenetic Remodeling**

**Hong Zhou^1^, Yujie Liu^1^, Geng G. Tian^2*^, Ji Wu^1, 3, 4*^**

1 Key Laboratory for the Genetics of Developmental and Neuropsychiatric Disorders (Ministry of Education), Bio-X Institutes, Shanghai Jiao Tong University, Shanghai 200240, China

2 School of Agriculture and Biology, Shanghai Jiao Tong University, Shanghai 200240, China

3 Key Laboratory of Fertility Preservation and Maintenance of Ministry of Education, School of Basic Medical Sciences, Ningxia Medical University, Yinchuan 750004, China

4 Lead Contact

*Correspondence: Geng G. Tian (gengtian@sjtu.edu.cn), Ji Wu (jiwu@sjtu.edu.cn)

**Supplementary Materials**

Figures S1 to S4

Tables S1 to S5

**Figure legends**

Figure S1 **Detection of H4K16ac protein levels in FGSCs treated with various concentrations of MC4033.** **(A)** Western blot analysis of H4K16ac protein expression. **(B)** Statistical analysis of H4K16ac protein levels. ***p* < 0.01, *****p* < 0.0001.

Figure S2 **Analysis of *Hmgb1* expression, cell viability, proliferation, and *Fyn* expression in *Hmgb1* knockdown cells following NMN co-treatment.** **(A)** qRT-PCR analysis of *Hmgb1* mRNA levels. **(B)** Western blot analysis and corresponding statistical results for HMGB1 protein levels. **(C)** Cell viability assay for *Hmgb1* knockdown cells after NMN co-treatment. **(D)** Representative images of EdU staining and the proportion of EdU-positive cells in *Hmgb1* knockdown cells following NMN co-treatment. **(E)** qRT-PCR analysis of *Fyn* mRNA levels. **(F)** Western blot analysis and corresponding statistical results for FYN protein levels. ns, *p* > 0.05.

Figure S3 **Impact of *Hmgb1* knockdown on chromatin accessibility in FGSCs.** **(A)** Heatmap showing TSS enrichment based on ATAC-seq data. **(B)** Visualization of peak distribution from ATAC-seq data. **(C)** Statistical analysis of peak distribution based on ATAC-seq data. **(D)** Volcano plot displaying differentially expressed peaks. **(E and F)** GO (E) and KEGG (F) pathway enrichment analyses of DEGs.

Figure S4 **Effects of *Hmgb1* knockdown on TAD structures and chromatin loops.** **(A)** Boxplot comparing Directional Index (DI) values before and after *Hmgb1* knockdown. **(B)** Fluctuation curves of insulation scores (IS) across TAD structures and adjacent genomic regions. **(C)** Heatmap of average observed/expected (O/E) Hi-C interactions within TAD regions pre- and post-*Hmgb1* knockdown. **(D)** Boxplot depicting changes in TAD boundary strength after *Hmgb1* knockdown. **(E)** Genome-wide analysis of intra-TAD interactions influenced by Hmgb1 knockdown. **(F)** Statistical analysis of chromatin loop counts. **(G)** Heatmap showing interactions within chromatin loops. **(H)** Boxplot quantifying contact frequencies within chromatin loops.

| Figure S1 |
| --- |
| 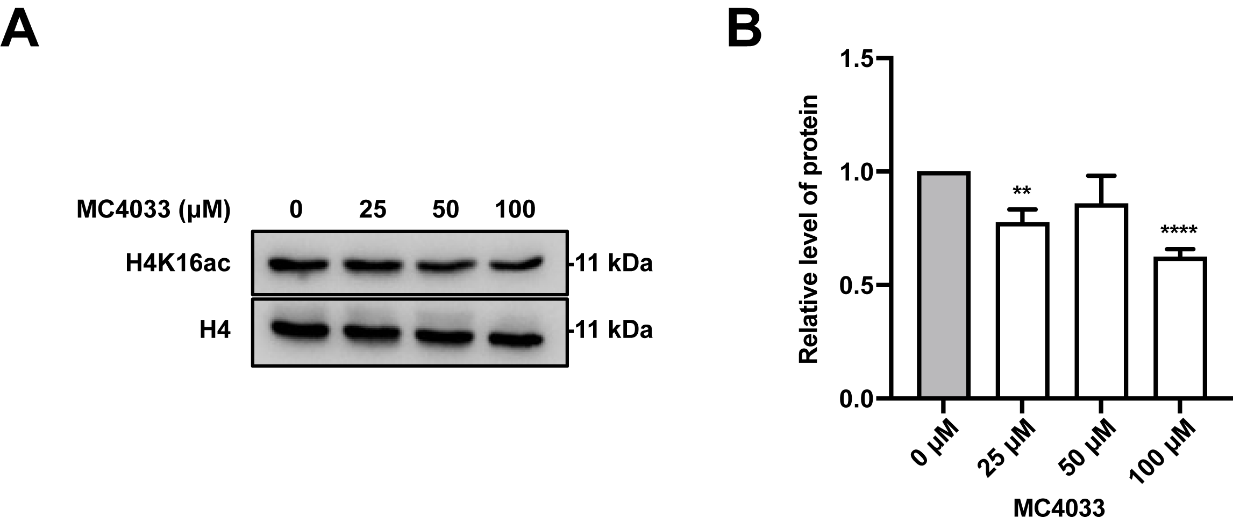 |

| Figure S2 |
| --- |
| 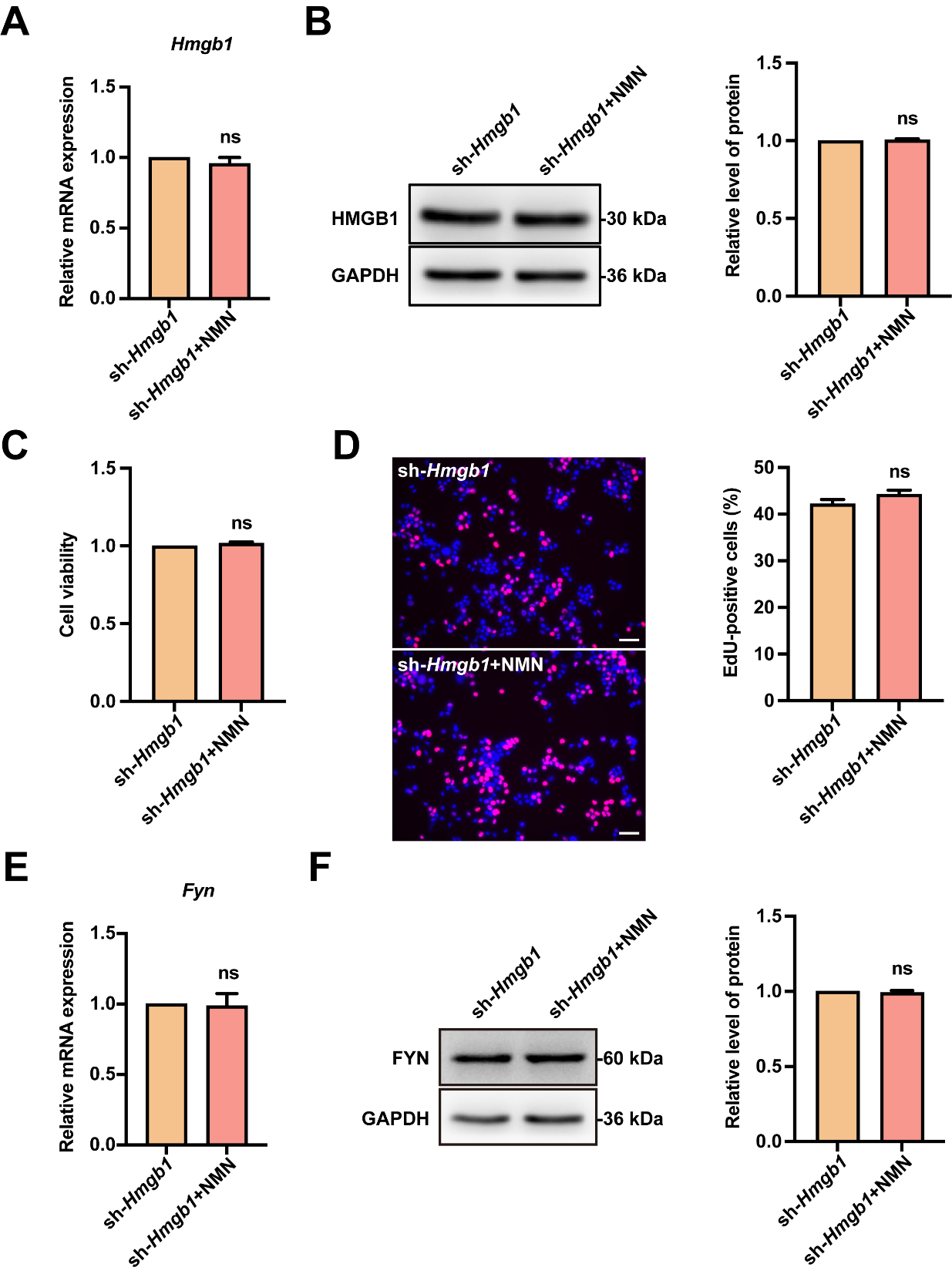 |

| Figure S3 |
| --- |
| 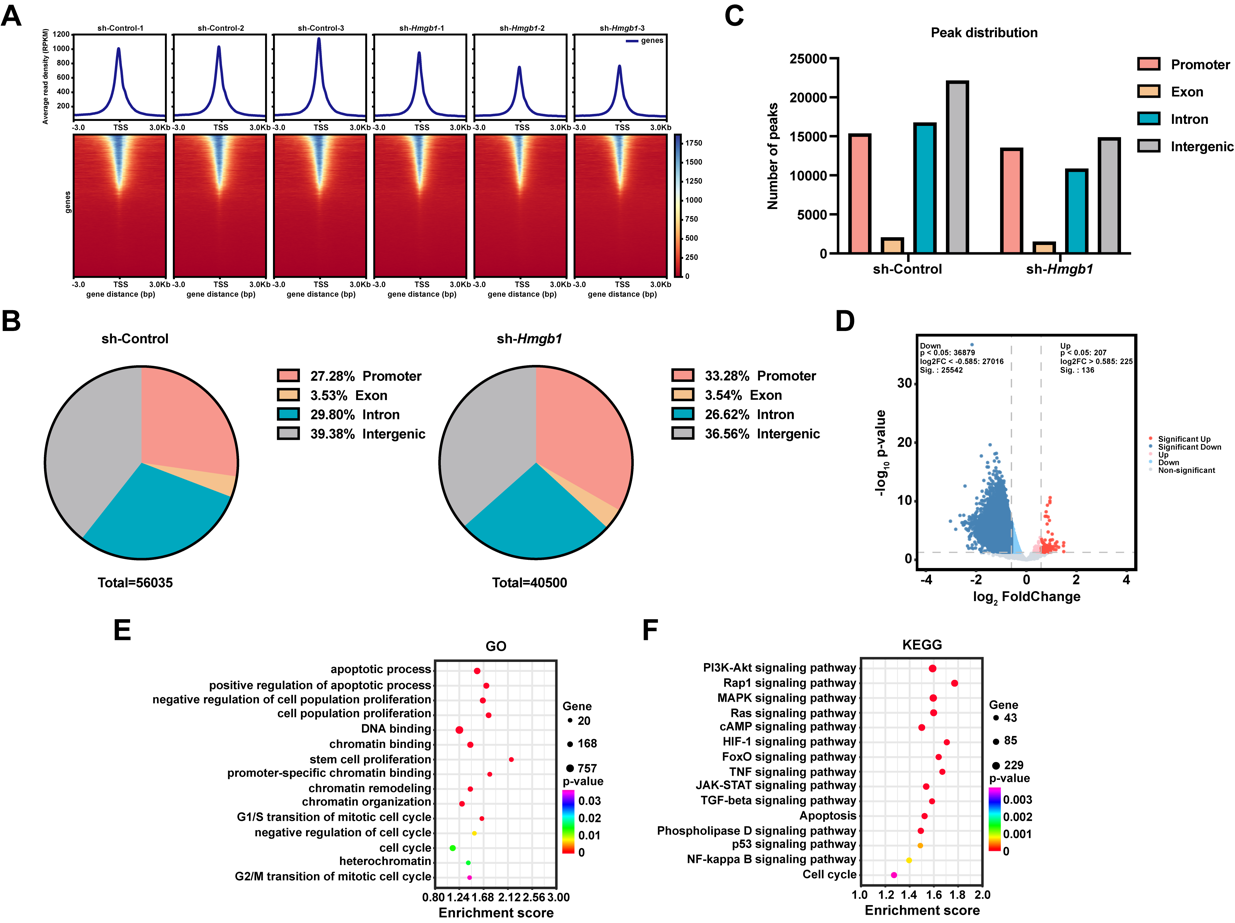 |

| Figure S4 |
| --- |
| 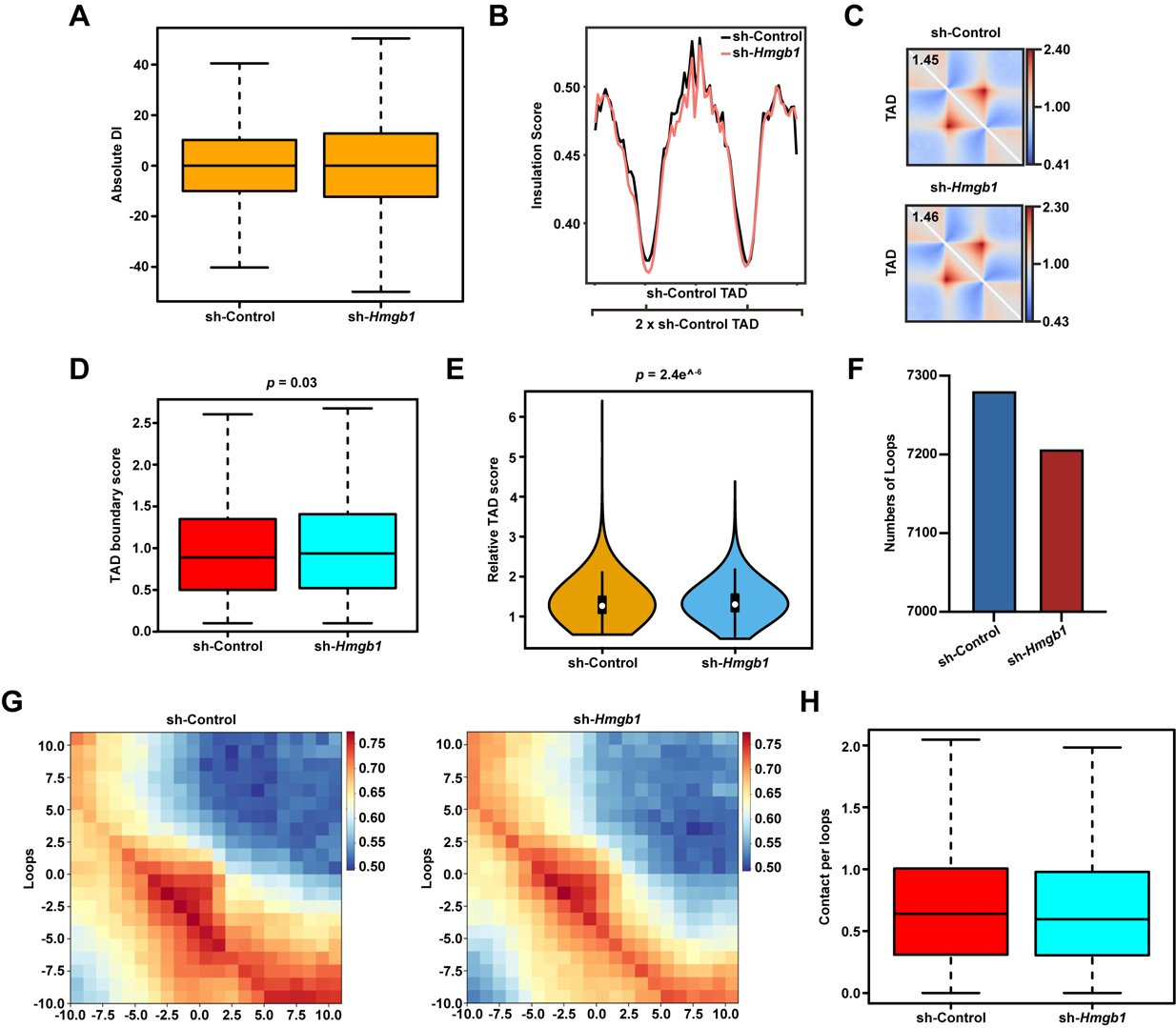 |

Table S1 Primer sequences used for ChIP-qPCR.

| Genes | Primer sequences |
| --- | --- |
| *Onecut3* promoter | F: CGAGCGAGAGCACTGAACAA  R: ATCCTGGGTGTCCTCTTCGT |
| *Tfdp1* promoter | F: GCGCAAATGCATACACACCT  R: CCCCACCTCTGCTCAACTAC |
| *Hmgb1* promoter | F: TGCAGCCCACCACAAGACA  R: GAAGAAGGGGTGCAACTGGA |
| *Plau* promoter | F: GCAGGATCCCTTAAGCAGCA  R: AGCCCTCCGCTGATACATTG |
| *Akt1* promoter | F: ACGCCCCATACCCTTCAGA  R: GCTGAGCCTCTGACTTAGCG |
| 1-*Fyn* promoter | F: AGAGCACTGCGGAAAAGACA  R: TTAGGCGCTCAAGGTCTGTG |
| 2-*Fyn* promoter | F: GGCAAAGTGGAAAGCAGCTC  R: TGTGAGAGGATGCGAGGTTG |
| 3-*Fyn* promoter | F: GCAGACCAACCCGGCTATTA  R: GCTCCTTGGGGCATTCAAAC |

Table S2 Primer sequences used for qRT-PCR.

| Genes | Primer sequences |
| --- | --- |
| *Onecut3* | F: TGAGCACACACGAAGAGGAC  R: CCACTAGGCTTCGGTGTTGG |
| *Tfdp1* | F:TTGAAGCCAACGGAGAACTAAAG  R: TGGACTGTCCGAAGGTTTTTG |
| *Hmgb1* | F: GGCGAGCATCCTGGCTTATC  R: GGCTGCTTGTCATCTGCTG |
| *Plau* | F: GCGCCTTGGTGGTGAAAAAC  R: TTGTAGGACACGCATACACCT |
| *Akt1* | F: ATGAACGACGTAGCCATTGTG  R: TTGTAGCCAATAAAGGTGCCAT |
| *Bax* | F: TGAAGACAGGGGCCTTTTTG  R: AATTCGCCGGAGACACTCG |
| *Bcl*-*2* | F: GTCGCTACCGTCGTGACTTC  R: CAGACATGCACCTACCCAGC |
| *Casp3* | F: ATGGAGAACAACAAAACCTCAGT  R: TTGCTCCCATGTATGGTCTTTAC |
| *PARP* | F: GGCAGCCTGATGTTGAGGT  R: GCGTACTCCGCTAAAAAGTCAC |
| *Zbtb32* | F: GGTACAGTTAGCGGCTAGACT  R: GGAAGGGCTTATGTCTTCAACC |
| *Fyn* | F: ACCTCCATCCCGAACTACAAC  R: CGCCACAAACAGTGTCACTC |
| *Cd28* | F: GTTCTTGGCTCTCAACTTCTTCT  R: TGAGGCTGACCTCGTTGCTAT |
| *Calr* | F: AAGATGCCCGATTTTACGCAC  R: CCCACAGTCGATATTCTGCTC |
| *Hspa5* | F: ACTTGGGGACCACCTATTCCT  R: ATCGCCAATCAGACGCTCC |
| *Wfs1* | F: CGGGAAGAAACGGACAGAGC  R: CGTAGGTAGTGTTTGCCCAC |
| *Egr1* | F: TCGGCTCCTTTCCTCACTCA  R: CTCATAGGGTTGTTCGCTCGG |
| *Gapdh* | F: AGGTCGGTGTGAACGGATTTG  R:TGTAGACCATGTAGTTGAGGTCA |

Table S3 shRNA sequences for *Hmgb1* knockdown.

| Genes | Sequences |
| --- | --- |
| sh-*Hmgb1*-1 | CGGCCTTCTTCTTGTTCTGTT |
| sh-*Hmgb1*-2 | TGACAAGGCTCGTTATGAAAG |
| sh-*Hmgb1*-3 | GATGACAAGCAGCCCTATGAG |
| sh-Control | CCTAAGGTTAAGTCGCCCTCG |

Table S4 Primer sequences for *Hmgb1* overexpression plasmid construction.

| Genes | Primer sequences |
| --- | --- |
| *Hmgb1*-F | CTT**AAGCTT**ATGGGCAAAGGAGATC |
| *Hmgb1*-R | CA**GAATTC**TTATTCATCATCATCATCTTCT |

Table S5 Primer sequences for dual-luciferase reporter vector construction.

| Genes | Primer sequences |
| --- | --- |
| *Fyn* promoter-F | G**CTCGAG**CCATCTCCCTTCCTTGCACA |
| *Fyn* promoter-R | C**CAAGCTT**TACTCTCGCTGATGCTCGGGCTGCT |
