# Supplementary figures and images for: Nicotinamide mononucleotide promotes female germline stem cell proliferation by activating the H4K16ac-Hmgb1-Fyn-PLD signaling pathway through epigenetic remodeling
Source: Cell Biosci. 2025 Apr 17;15:48. doi: 10.1186/s13578-025-01387-w (PMC12004683; doi:10.1186/s13578-025-01387-w)

2A

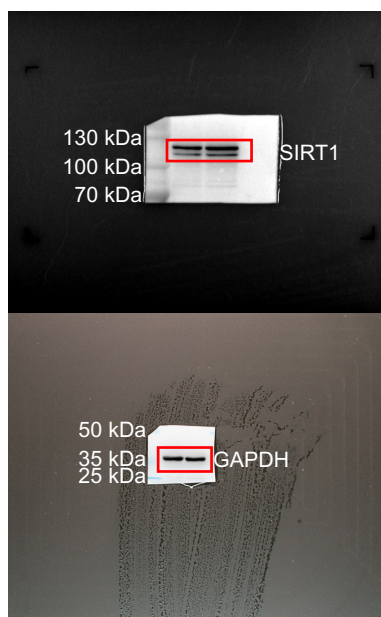

2B

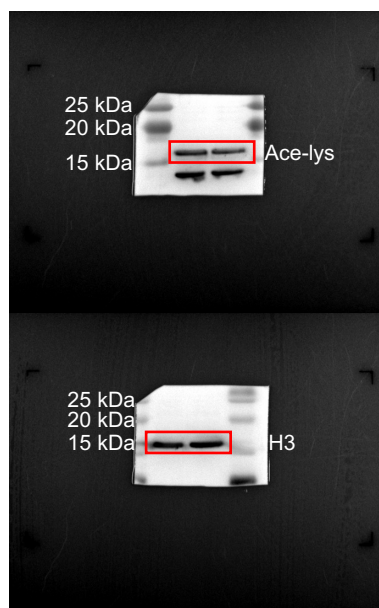

2I

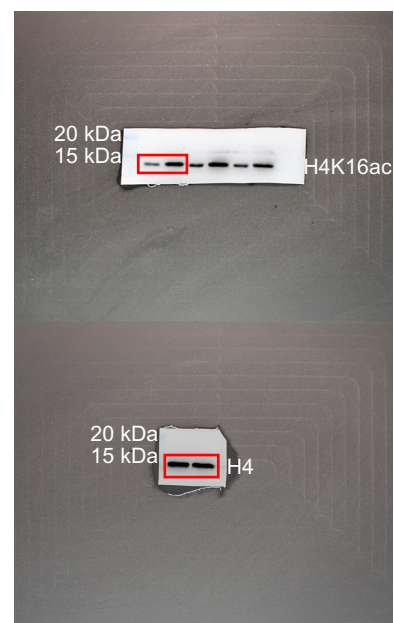

2K

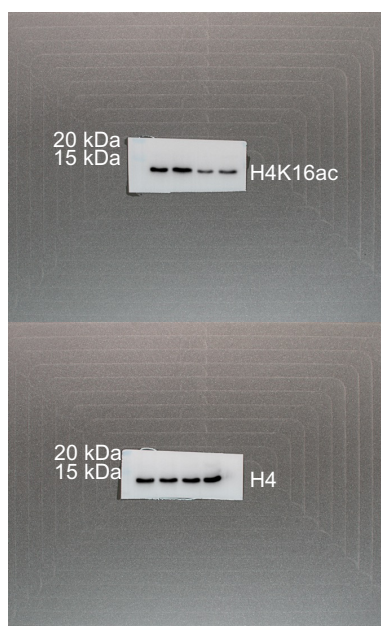

4E

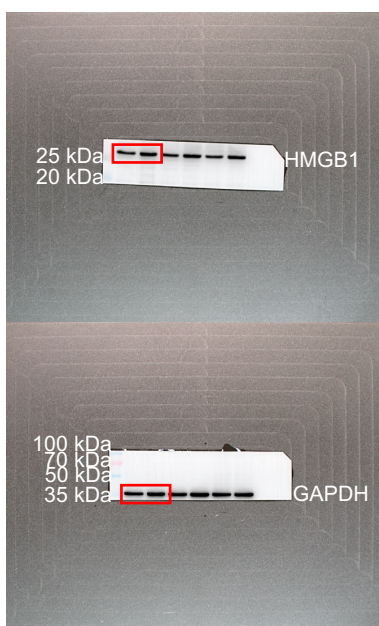

4G

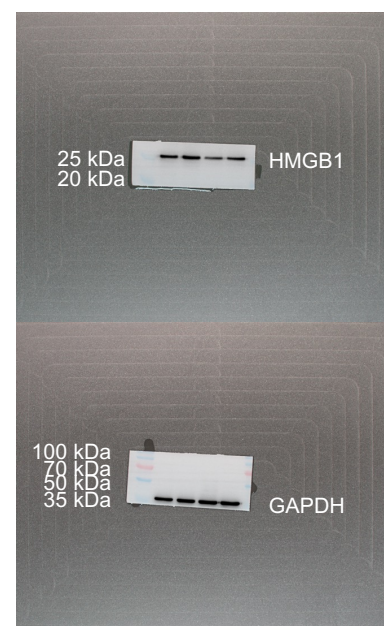

4I

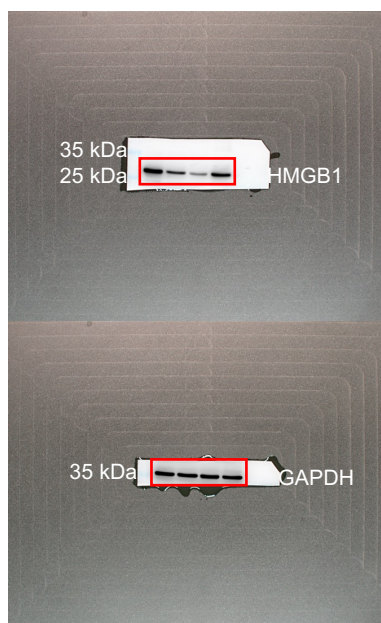

S1A

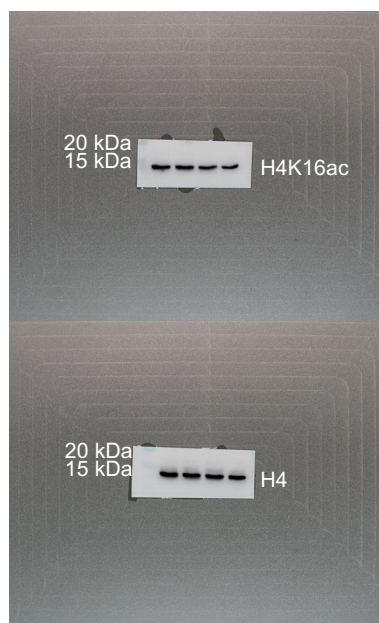

S2B

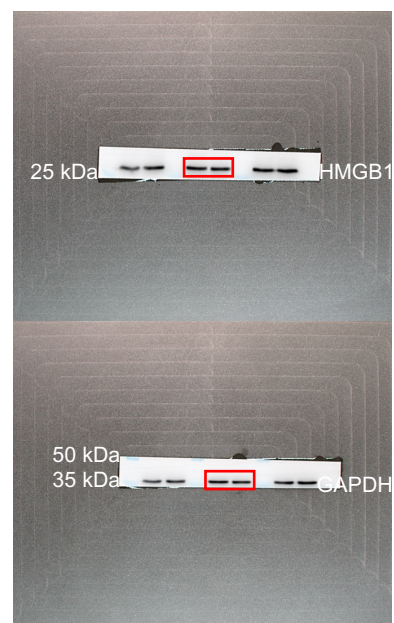

5G

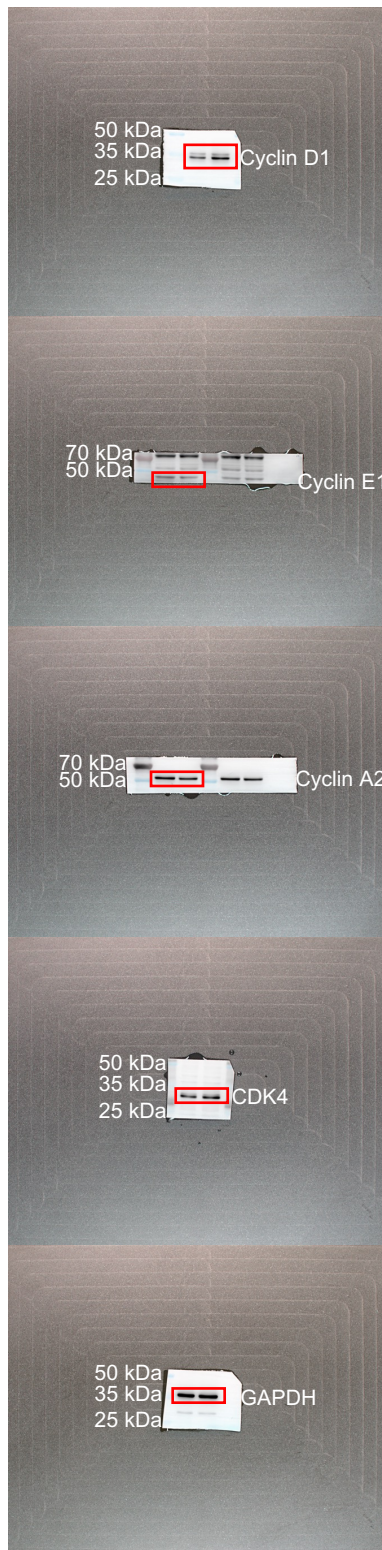

5K

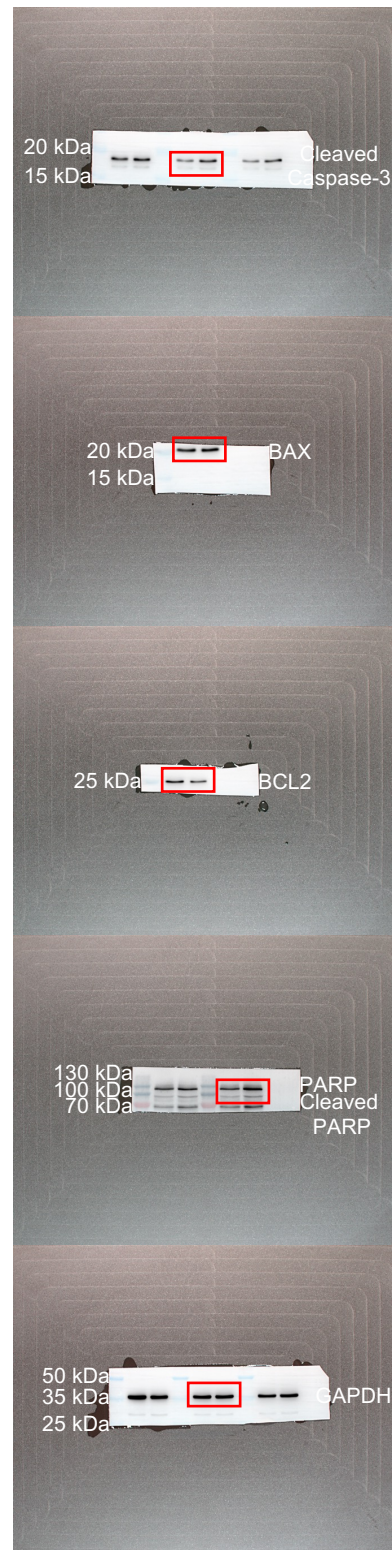

7E

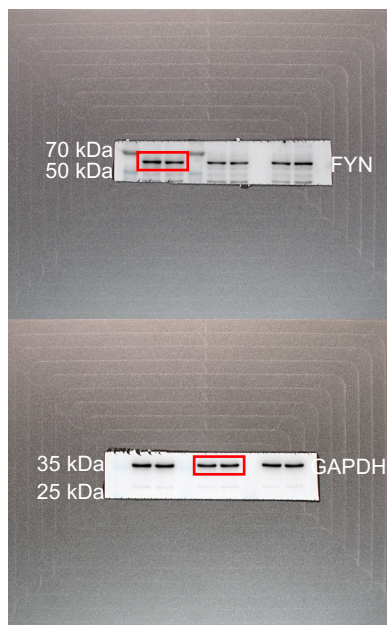

7H

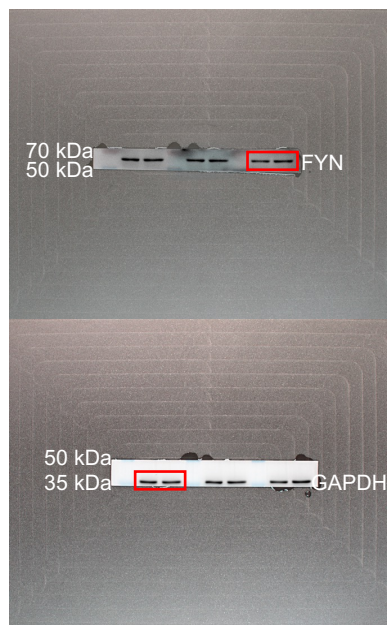

8E

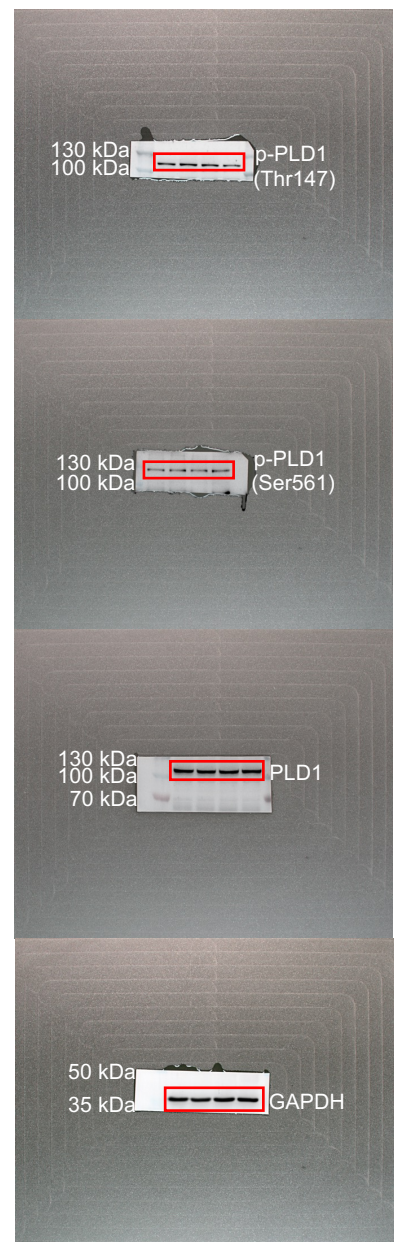

7J

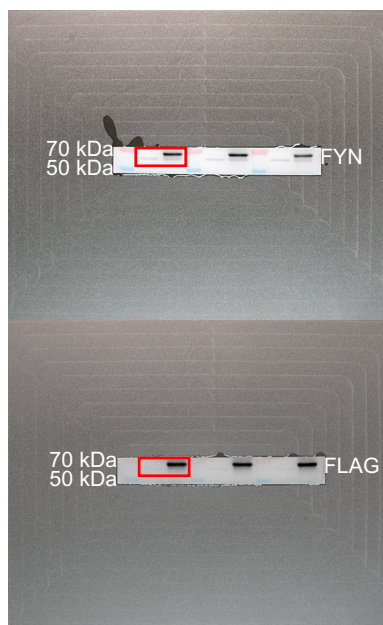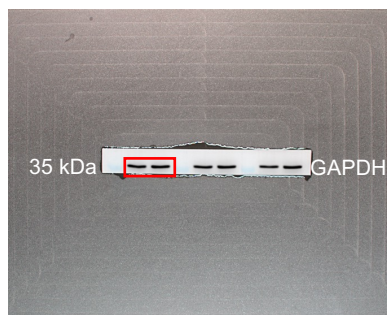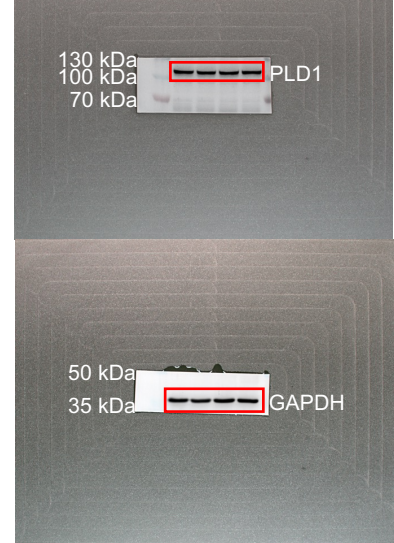

Supplement: Supplementary file 2 — Additional file 2 contains the full uncropped blot images. [file 13578_2025_1387_MOESM2_ESM.pdf]
